# Proteomic Analysis Reveals the Protective Effects of Yiqi Fumai Lyophilized Injection on Chronic Heart Failure by Improving Myocardial Energy Metabolism

**DOI:** 10.3389/fphar.2021.719532

**Published:** 2021-09-21

**Authors:** Xiaoying Han, Yi Zhang, Ou Qiao, Haixia Ji, Xinyu Zhang, Wenzhe Wang, Xia Li, Juan Wang, Dekun Li, Aichun Ju, Changxiao Liu, Wenyuan Gao

**Affiliations:** ^1^ School of Pharmaceutical Science and Technology, Tianjin University, Tianjin, China; ^2^ Tasly Pride Pharmaceutical Company Limited, Tianjin, China; ^3^ Tianjin Pharmaceutical Research Institute, Tianjin, China

**Keywords:** chronic heart failure, Yiqi Fumai lyophilized injection, oxidative phosphorylation, mitochondrial biogenesis, perixisome proliferation-activated receptor alpha

## Abstract

Yiqi Fumai lyophilized injection (YQFM) is the recombination of Sheng mai san (SMS).YQFM has been applied clinically to efficaciously and safely treat chronic heart failure (CHF). However, the mechanism of YQFM is still not fully elucidated. The purpose of our study was to investigate the protective mechanism of YQFM against abdominal aortic coarctation (AAC) in rats by proteomic methods. After YQFM treatment, the cardiac function were obviously meliorated. One hundred and fifty-seven important differentially expressed proteins (DEPs) were identified, including 109 in model rat compared with that in control rat (M:C) and 48 in YQFM-treated rat compared with that in model rat (T:M) by iTRAQ technology to analyze the proteomic characteristics of heart tissue. Bioinformatics analysis showed that DEPs was mainly involved in the body’s energy metabolism and was closely related to oxidative phosphorylation. YQFM had also displayed efficient mitochondrial dysfunction alleviation properties in hydrogen peroxide (H_2_O_2_)-induced cardiomyocyte damage by Transmission Electron Microscope (TEM), Metabolic assay, and Mitotracker staining. What’s more, the levels of total cardiomyocyte apoptosis were markedly reduced following YQFM treatment. Furthermore, Western blot analysis showed that the expressions of peroxisome proliferator activated receptor co-activator-1α(PGC-1α) (*p* < 0.01 or *p* < 0.001), perixisome proliferation-activated receptor alpha (PPAR-α) (*p* < 0.001)and retinoid X receptor alpha (RXR-α) were upregulated (*p* < 0.001), PGC-1α as well as its downstream effectors were also found to be upregulated in cardiomyocytes after YQFM treatment(*p* < 0.001).These results provided evidence that YQFM could enhance mitochondrial function of cardiomyocytes to play a role in the treatment of CHF by regulating mitochondrial biogenesis-related proteins.

**Graphical Abstract:**
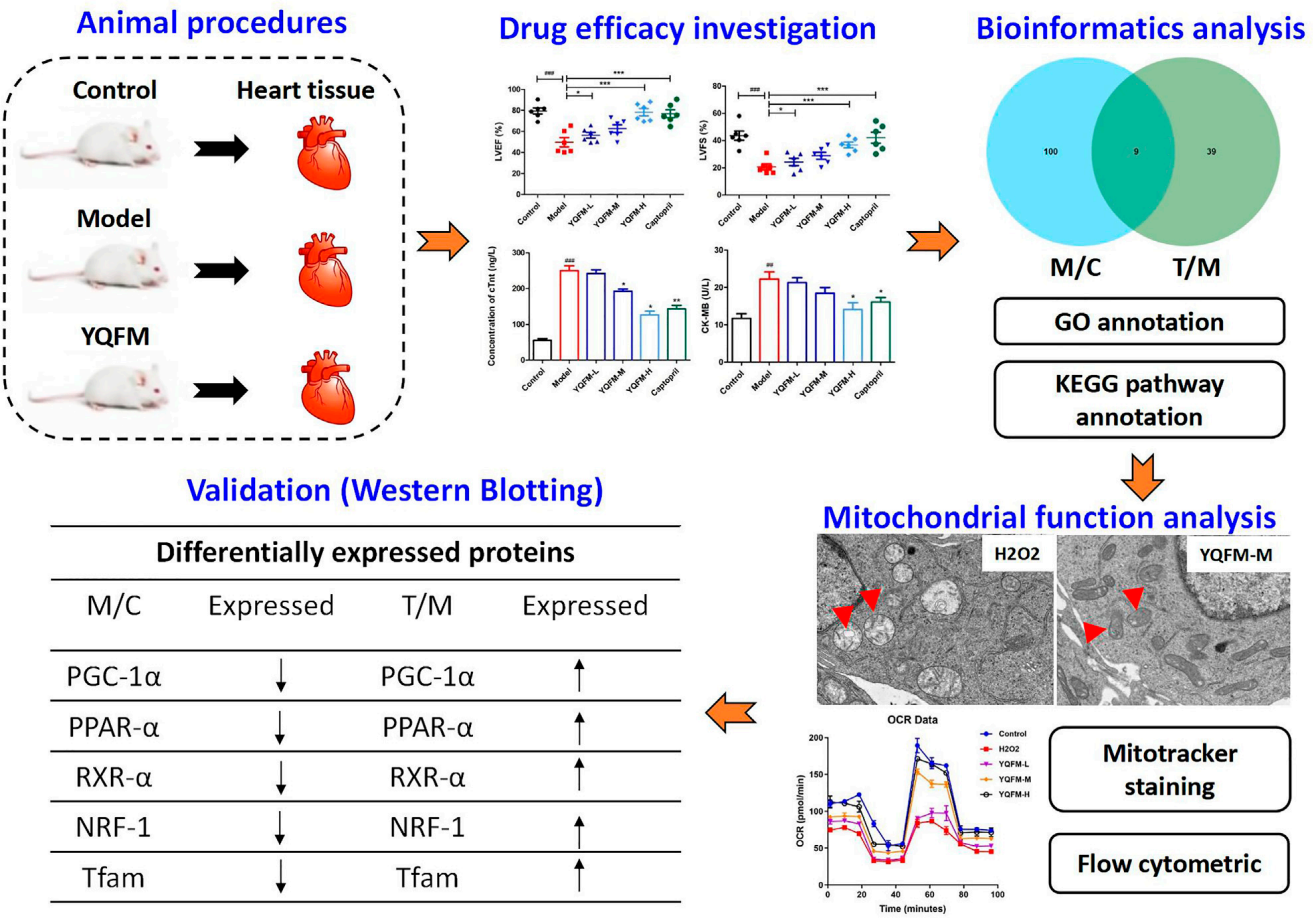
Potential mechanism of YQFM in the treatment of chronic heart failure is to improve myocardial energy metabolism via enhancing the mitochondrial function of myocardial.

## Background

Chronic heart failure is myocardial damage caused by various reasons, resulting in injures the function and structure of the myocardium, and finally resulting in low ventricular pumping or filling function (Mosterd and Hoes, 2007). The mortality rate of patients with CHF(4-year) is up to 50%, and the mortality rate of patients (1-year) with severe heart failure is as high as 50% (Heo et al., 2008; Yancy et al., 2013; Wang et al., 2017). Therefore, CHF is an important challenge facing the cardiovascular field in the 21st century. With the deepening of the understanding of the pathogenesis of heart failure, Western medicine’s treatment of CHF has gradually shifted from a cardiotonic, diuretic, and vasodilator-based cardiac circulation model to prevent ventricular remodeling, improve prognosis, and recover. Targeting the synchrony of ventricular function, these drugs can alleviate patient mortality and relieve symptoms with advantage. However, there are some adverse events that may limit the use of TCM (Fu et al., 2010; Faris et al., 2012; Wang et al., 2015a), so conventional treatment of western medicine is often combined with TCM syndrome differentiation and treatment, and the advantages of Chinese and Western medicine are complemented.

YQFM derives from the classic name “Sheng Mai SAN,” made by Radix of Panax ginseng C.A. Mey. (Araliaceae), Fructus of Schisandra chinensis (Turcz.) Baill (Schisandraceae), and Radix of *Ophiopogon japonicus* (L.f) Ker-Gawl. YQFM is a TCM powder injection produced by Tasly Pharmaceutical Co., Ltd., It is mainly used for coronary heart disease fatigue angina pectoris syndrome of Qi and Yin deficiency, and chronic left ventricular insufficiency caused by coronary heart disease II and III Qi and Yin deficiency. Since its official launch in 2007, the quality is stable and the curative effect is accurate, and it has been wide-ranging recognized by doctors and patients. Past studies have shown that its active ingredients could melioration HF by inhibiting the activity of NF-κB (Xing et al., 2013), and reduce hypoxia-induced myocardial injury (Feng et al., 2016; Li et al., 2016). In addition, YQFM can regulate MAPKs and alleviate myocardial remodeling and heart failure caused by coronary artery ligation (CAL) (Pang et al., 2017). Many components of YQFM show mitochondrial regulation (Zheng et al., 2018), including ginsenosides Rb1, Rb3, Rg1, Rg3, Schisandra B, and ophiopogon D (Chiu et al., 2007; Chen and Ko, 2010; Sun et al., 2013; Mu et al., 2015; Dong et al., 2016; Chen et al., 2019; Park et al., 2019; Li et al., 2021). Nevertheless, the mechanism of YQFM in treating chronic heart failure remains to be further elucidated. Differential proteomics is a method to clarify the pathological changes of diseases or the mechanism of therapeutic intervention through differential expression analysis of proteins (Wilhelm et al., 2014), to obtain “panoramic information” about life activities within a short period. Differential proteomics analyzes the dynamic evolution process of protein expression in the body under different conditions from a holistic perspective. Its research method is very consistent with the method of TCM syndrome differentiation and treatment, and the comprehensive regulation of TCM prescriptions. With the help of the development and breakthrough of proteomics technology, it is now possible to study the changes in the overall protein expression of various organs in the organism under different internal and external interventions and influences, such as in the onset of cardiovascular disease, and to detect the changes in the cell The composition, expression and regularity of this protein (Suo et al., 2016; Wei et al., 2019). The application of proteomics to the research of TCM can effectively help researchers to find the targets of TCM compounds, analyze the biological processes involved in the pharmacological effects of TCM, and further explain the mechanism of TCM from the perspective of molecular biology.

This study was aimed to explore the therapeutic effect and mechanism by which YQFM attenuates AAC-induced CHF in rats and H_2_O_2_-induced cardiomyocyte damage in H_9_C_2_ cells. However, due to the complexity of TCM prescriptions and various causes of CHF, the mechanism of the optimized prescription is not very clear. Therefore, this experiment used differential proteomics to investigate the targets protein regulated by YQFM in treating CHF rats, and combined with biological function analysis to explore the possible biological basis of the therapeutic effect of the formula. Western blot verification were used to confirm that the mechanism of YQFM in the treatment of CHF.

## Materials and Methods

### Chemicals and Materials

YQFM was donated by Tasly Pride Pharmaceutical Company Limited. Lactate dehydrogenase (LDH) assay kit, rat cardiac troponin-T (cTnT) and creatine kinase isoenzyme-MB (CK-MB) ELISA kits were obtained from Nanjing Jiancheng Bioengineer Institute. Antibodies against cleaved cTnT, Bax, Bcl-2, PGC-1α, PPAR-α, RXR-α, Tfam and NRF-1 and secondary anti-rabbit antibodies were purchased from Abcam, (Cambridge, United Kingdom). Hydrogen peroxide (H_2_O_2_, Aladdin®, 700 μmol/L).

### HPLC-QQQ-MS/MS Analysis Condition

Sample solutions for qualitative analysis. 2.60 g of YQFM was accurately weighed and dissolved in 30% methanol (10 ml). Sample solution was added into C8 solid-phase extraction (SPE). The samples were eluted in turn with the following solutions: 30% methanol solution containing 2 ml sodium hydroxide (0.5 mol/L), 30% methanol (5 ml) and 100% methanol (5 ml). The 100% methanol eluent was collected and diluted to 5 ml. The obtained test solution should be filtered through a 0.22 mm syringe filter. Accurately weigh the ginsenosides Rb1, Rf, Re, Rc, Rb2, Rb3, Rg1, Rh1, Rc, Rd, Rf2, Rg2, Schisandrin A, Schisandrin B, Schisandrin A, and Schisandrin B reference substance, add 50% Methanol is dissolved, and the mass concentration of ginsenoside Rb1, Rf, Re, Rc, Rb2, Rb3, Rg1, Rh1, Rc, Rd, Rf2, Rg2, schisandrin A, and schisandrin B are each 20 μg/ml. A mixed reference solution of ethyl alcohol and schisandrin A 10 μg/ml.

Sample solutions for qualitative analysis. Chemical analysis was performed on high performance liquid chromatography (HPLC) system. Two mobile phases were used for chromatographic separation on kromasil 100-5-C18 column (5 μm, 4.6 mm × 250 mm): Phase A is ultrapure water with 0.1% formic acid and Phase B is acetonitrile. Gradient elution program: 0–8 min, 20–30% B; 8–10 min, 30–32% B; 10–15 min, 32–35% B; 15–26 min, 35–35% B; 26–35 min, 35–40% B; 35–40 min, 40–45% B; 40–50 min, 45–55% B; 50–60 min, 55–70% B; 60–65 min, 70–95% B; 65–72 min, 95–98% B; 72–81 min, 98–20% B. When the injection volume was 10 μl, the flow rate: 1 ml/min, and the column temperature was 28°C.

### Model Establishment

Sprague-Dawley (SD) male rats weighing (220–240) g were obtained from the Beijing HFK Biotechnology Co., Ltd (SCXK-2018-0004). All the rats were then subjected to 1 week of domestication before experiment. The rats were kept in a 22 ± 2°C cage with light/dark circulation for 12 h and humidity of 40 ± 5%. In addition, the rats were fed rat chow and given free water.

Abdominal aortic coarctation (AAC) establishes a pressure overload CHF model. Its mechanism is that by narrowing the abdominal aorta, aortic pressure increases, cardiac afterload increases, myocardial compensatory hypertrophy, ventricular volume increases, heart expands, cardiac decompensation in the later stage, resulting in myocardial function and structural damage, and finally heart failure. AAC model in rats was established by referring to the reference method (Cops et al., 2019). The steps were as follows. After intraperitoneal injection of 2% sodium pentobarbital at 0.2 ml/100 g (Sigma-Aldrich, St. Louis, MO, United States) and preparation of the skin (shaved), the rat was fixed on the operating table supine. The abdominal aorta above the renal artery branch was blunt dissociated. The needle of No. 7 syringe was parallel to the abdominal aorta, and No. 4 non absorbable surgical silk thread was used to connect the abdominal aorta and syringe. The needle was ligated together, and then the syringe was slowly withdrawn, the abdomen was closed, and layered sutures were used to narrow the diameter of the rat’s abdominal aorta to 0.7 mm, and then 0.1 ml of penicillin was injected into the abdominal cavity to prevent infection.

### Animal Grouping and Administration

Except for 10 rats in the blank control group, 55 of the 70 rats were made into AAC models. They were then grouped randomly into control, AAC, AAC+YQFM low-dose (YQFM-L, 20 mg/kg/d) arms, AAC+YQFM medium-dose (YQFM-M, 40 mg/kg/d) arms, AAC+YQFM high-dose (YQFM-H, 80 mg/kg/d) arms and captopril (Capt., 40 mg/kg/d) group. The dose of rats in the middle group received an adult equivalent dose of 70 kg, the dose of rats in the high group received twice the adult equivalent dose, and the dose of rats in the low group received half of the adult equivalent dose.

After 4 weeks of intervention, the rats were anesthetized. 2% sodium pentobarbital (Shanghai, China) (0.2 ml/100 g) was injected intraperitoneally. The blood was gathered from the femoral artery, centrifuged and stored at −80°C for analysis. After perfusing the heart with cold PBS to remove blood, filter paper was used to remove free PBS. The heart tissue was immersed with 4% paraformaldehyde (Google Biotechnology, Wuhan, China), and a biopsy was performed to monitor the morphology of the myocardial tissue. The remaining heart tissue was saved in liquid nitrogen for 1 h and stored at −80°C before proteomics and Western blot analysis.

### Echocardiography

Four weeks after administration, the rats were anesthetized with 2% sodium pentobarbital and underwent echocardiography on an ultrasound machine. Left ventricular posterior wall thickness (LVPWd, LVPWs) during diastolic and systolic periods was recorded in M mode to examine left ventricular thickening (Luo et al., 2015).

### Biochemical Parameters

cTnT, LDH and CK-MB in serum are unique biomarkers of myocardial injury after heart failure (Bertinchant et al., 2000; Giannitsis and Katus, 2013). After 28 days, the serum CK-MB and LDH were detected by blood biochemical examination to evaluate the cardiomyocyte repair. Quantitative examination of cTn T and CK-MB and LDH analysis on serum samples from different research groups using assay kits.

### Cardiac Histopathological Examination

The left ventricular region of each group was fixed in 4% paraformaldehyde for 24 h. After fixation, it was embedded with paraffin and stained with hematoxylin and eosin (HE), Masson and Periodic Acid Schiff (PAS) respectively.The pathological changes of myocardial fibers in each group of rat were observed by an optical microscope (JEOL, Tokyo, Japan).

### Immunohistochemical Measurement of cTnt

Immunohistochemical analysis of cTnt expression in the heart. Paraffin-embedded tissue was sectioned continuously at 4 μm, dewaxed with xylene and hydrated with gradient alcohol. The sections were incubated with goat serum (10%) for 1 h, then combined with primary antibody and incubated overnight at 4°C. Follow the instructions in the manufacturer's agreement for subsequent procedures. The Image Pro Plus software was used to evaluate images.

### Protein Extraction and Tandem Mass Tags Protein Labelling

Randomly collect rat myocardial tissue, three samples in control arm, three samples in model arm, and three samples in the YQFM-H treatment group. All frozen samples were took out and ground them with liquid nitrogen. Then the same amount of samples was transferred to MP shaker tubes, and moderate dose of protein lysis solution (1% SDS + 8 M urea, containing protease inhibitors) was added. FastPrep®-24 instrument (MP Biomedicals, OH, United States) was used to homogenize heart tissue samples in pyrolysis buffer (TEAB, 25 mM Triethylammonium bicarbonate, 2% sodium dodecyl sulfate (SDS)). The protein was quantified according to the BCA kit instructions. Samples from each animal in the group were then divided into equal groups. Tissue samples, 50 μg of reference cell containing the same amount of all samples and 50 μg of total protein in each combination group, were reduced by DL-Dithiothreitol and then trypsin was digested using the membrane assisted sample preparation method modified by Wiśniewski et al. (2009).The sample diluted with 8 M urea was used to filter (Nanosep 30 k Omega, Pall Life Sciences, Washington, New York, United States), and the SDS was washed repeatedly with 8 M urea.

Methyl methane thiosulfonate diluted in digestion buffer (20 mM TEAB, 1% sodium deoxycholate (SDC)) was used for alkylation, and the membrane was washed repeatedly with digestion buffer. Trypsin was added to 25 mM TEAB at a ratio of 1:100 relative to protein mass, and the samples were incubated overnight at 37°C. The next morning, another portion of trypsin was added and the samples were incubated at 37°C for 4 h (Boersema et al., 2009; Xu et al., 2014; Liu et al., 2017). According to the manufacturer’s instructions, the peptide was labeled with isobaric mass labeling reagent TMT®. In one group, each reference and sample were labelled with a unique label from the TMT 6 plex or 10 plex isobaric mass labelling kit. After TMT labeling, a set of samples were combined, concentrated and acidified to about pH 2 to precipitate SDC.

### High Performance Liquid Chromatography Fractionation

Trypsin samples were separated into fractions by high-pH reverse HPLC with ACQUITY UPLC BEH C18 (1.7 μm particles, 2.1 mm ID, and 250 mm length) (Wang et al., 2010; Song et al., 2011). The procedure was as follows: Within 48 min, a gradient of 0–100% acetonitrile (pH = 10.0) was used to separate peptides. According to the time and peak type, 20 fractions were collected for each group, and they were combined into 10 fractions. After vacuum centrifugation, they were dissolved in mass spectrometry loaded buffer for two-dimensional analysis.

### LC-MS/MS Analysis

Tryptic peptide was dissolved in 0.1% (V/V) formic acid, and then 9RKFSG2_NCS-3500RS (Thermo, United States) Ultra High Performance Liquid System was used for separation (Motoyama and Yates, 2008). 0.1% formic acid and 2% acetonitrile were contained in Solvent A, while 0.1% formic acid and 80% acetonitrile were contained in solvent B. Gradient elution: 0–6 min, 0–7% solvent B; 7–68 min, 7–24% solvent B; 69–80 min, 24–29% solvent B; 80–90 min, 29–39% solvent B; 90–94 min, 39–52% solvent B; 94–97 min, 52–100% solvent B; 97–105 min, 100–100% solvent B; and 105–106 min, 0% solvent B. The flow rate was kept at 300 nl/min.

The peptides has passed a nano-electrospray ionization source and then analyzed by Q-Exactive Plus(Thermo, United States)mass spectrometry.The resolution of the primary mass spectrometer is 70,000, AGC target 3e6, the fragmentation method was HCD, and maximum injection time is 20 ms; the secondary resolution was 35,000, AGC target 1e5, the maximum injection time was 50 ms. In the MS investigation scan, the first 20 precursor ions above the threshold ion count 5E4 were scanned once, and then the alternate data dependence program between 20 ms/MS scans was performed, and 18.0 s was excluded dynamically. Automatic gain control was used to prevent the ion trap from being over full. m/z scan range: 350–1300.

### Database Search

The software version used by the database was Proteome Discoverer ^TM^ Software 2.2. When searching the database, the original file has been submitted to the Proteome Discoverer server, and then the database was searched in the established database.

### Bioinformatics Analysis

Gene Ontology (GO) annotated proteome comes from Genebank database. Then, through GO annotation, proteins were classified according to biological process, cell composition and molecular function. The KEGG database was accustomed to explain the pathways of identified proteins.

### Cell Culture and Treatment

H9C2 cells were obtained from the Chinese Academy of Sciences (Shanghai, China). Dulbecco’s modified eagle’s medium (DMEM) containing 10% (v/v) FBS, 1% (V/V) penicillin-streptomycin solution was used for cell culture, and placed in the incubator at 37°C with a humidified 5% CO_2_. To stimulate cardiomyocyte injury, H_2_O_2_ was used to stimulate H_9_C_2_ cells. H_9_C_2_ cells were treated with 45 µg/ml (YQFM-L), 90 µg/ml (YQFM-M) or 180 µg/ml (YQFM-H) YQFM for 24 h.

### Transmission Electron Microscopy

H9C2 cells were inoculated on 6-well plates and H_2_O_2_ was used to cause myocardial cell damage. After 24 h, the cells were treated with YQFM (90 µg/ml) for 24 h. The cultured cells were fixed on 2.5% glutaraldehyde for 1 h respectively with glutaraldehyde, dehydrated, critical-point dried, metal sputtering, and analyzed by TEM at 6,000 × magnification.

### Metabolic Assay

H9C2 cells were inoculated in 96-well plates. Except for control group, H_2_O_2_ was used to simulate myocardial cell injury in other groups. After 24 h, cells were treated with different dose of YQFM (45, 90, and 180 µg/ml) for 24 h. DMEM medium was replaced with 100 μl XF containing 4,500 mg/L glucose. The cells were then incubated in the absence of CO_2_ at 37°C for 1 h. According to the manufacturer’s agreement, oxygen consumption rate (OCR) was measured by SeaHorse XF96 Extracellular Analyzer as a measure of oxidative metabolism.

### Immunofluorescence Staining

After the indicated treatments, H_9_C_2_ cells were seeded in chamber slides at a density of 1 × 10^6^ cells/well. Take a slice of cardiomyocytes that have been cultured for 24 h. After treated with or without YQFM for 24 h, immunostaining of the cells was carried out with Mitotracker (Thermo Fisher Scientific, Waltham, MA, United States). Incubate with DAPI staining solution for 10 min, and wash with PBS three times; immediately after mounting with anti-fluorescence quenching solution, observe under a confocal microscope.

### Apoptosis Assay

FITC-labeled annexin V and propidium iodide staining were used to evaluate the apoptosis of the treated cells, and FACS calibur flow cytometry (Becton Dickinson, United States) was used to examine the level of apoptosis. The cell cycle was subsequently checked using FlowJo software.

### Western Blot Analysis

The left ventricular protein was extracted with RIPA lysate containing protease inhibitors, and the protein concentration was determined by BCA method. The SDS-PAGE was performed, electrophoresed to nitrocellulost membrane, and blocked with 5% bovine serum albumin for 1 h. Next, antibodies and dilutions as follows: Bax (Abcam, ab32503, rabbit), Bcl-2 (Abcam, ab182858, rabbit), PGC-1α (Abcam, ab176328, rabbit), PPAR-α (Abcam, ab245119, rabbit), RXR-α (Abcam, ab125001, rabbit), NRF-1 (Abcam, ab55744, rabbit) and Tfam (Abcam, ab176558, rabbit).The fluorescently labeled secondary antibodies are then conjugated by incubation. The blotted proteins were examined and quantified on the Odyssey Infrared Imaging system. The ratio of the target protein to the internal reference protein β-actin reflects the relative expression of the protein.

### Statistical Analyses

All data are expressed as the means ± SD. GraphPad Prism statistical software was used for analysis, and one-way analysis of variance was used for comparison between groups; *p* < 0.05 was considered statistically significant.

## Results

### Analysis of Chemical Components of Yiqi Fumai Lyophilized Injection

**FIGURE 1:**
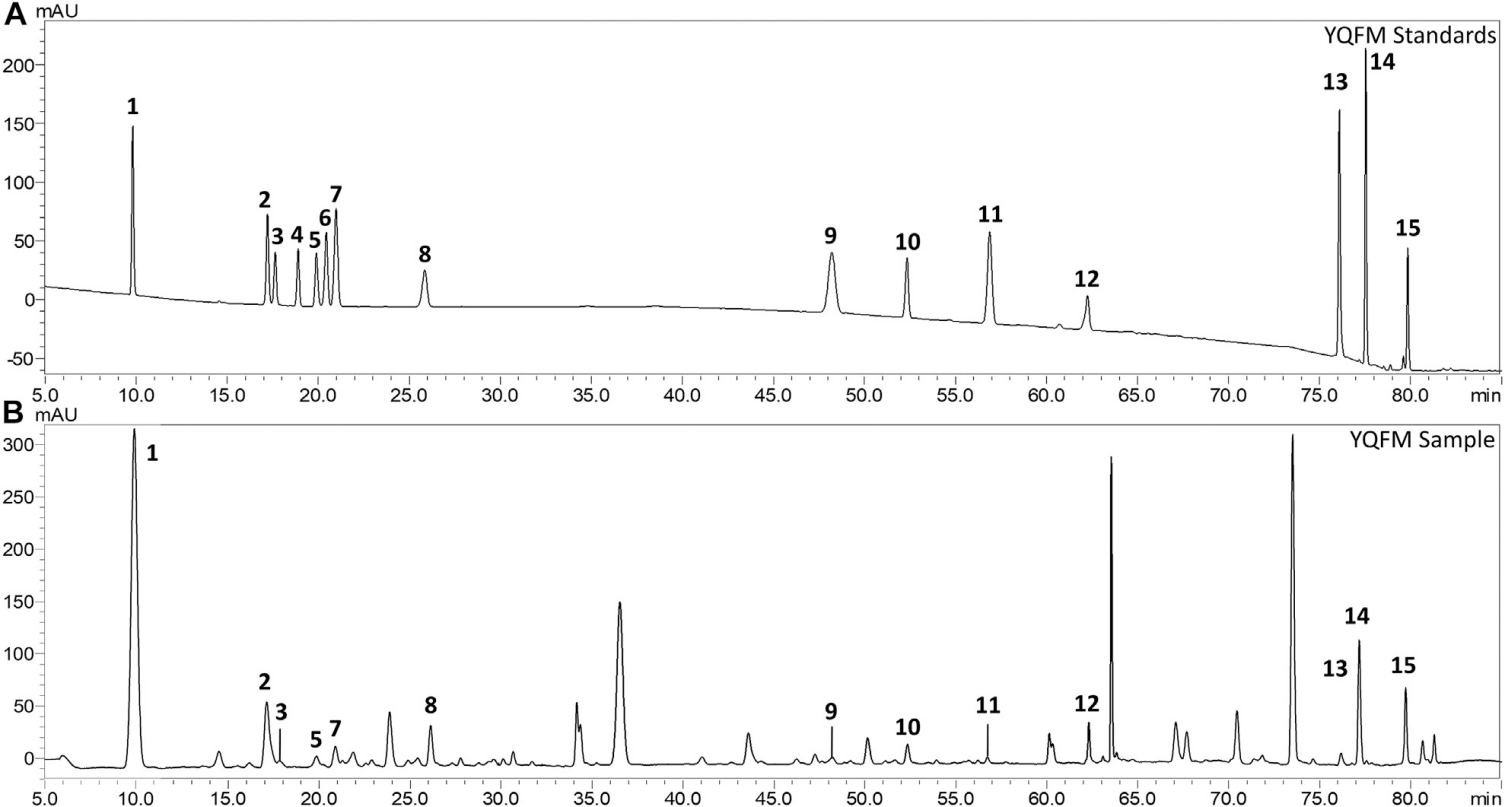
Comparison of the liquid chromatogram of the mixed reference substance and YQFM sample. **(A)** Standard solution and **(B)** Sample solution: 1: ginsenoside Rg1, 2: ginsenoside Rf, 3: ginsenoside Rb1, 4: ginsenoside Rc, 5: ginsenoside Rb2, 6: ginsenoside Rb3, 7: ginsenoside Re, 8: ginsenoside Rd, 9: schizandrol A, 10:20(S)-ginsenoside F2, 11: schizandrol B, 12: ginsenoside Rg2, 13: schisandrin A, 14: schisandrin B, 15: ginsenoside Rh1.

In order to detect the chemical constituents of YQFM, an HPLC method was established. After analyzing the HPLC of YQFM standard solution and sample solution (Figures 1A,B). A total of 14 compounds were identified from YQFM.

### Effects of Yiqi Fumai Lyophilized Injection on Cardiac Dysfunction in Chronic Heart Failure Rat

**FIGURE 2:**
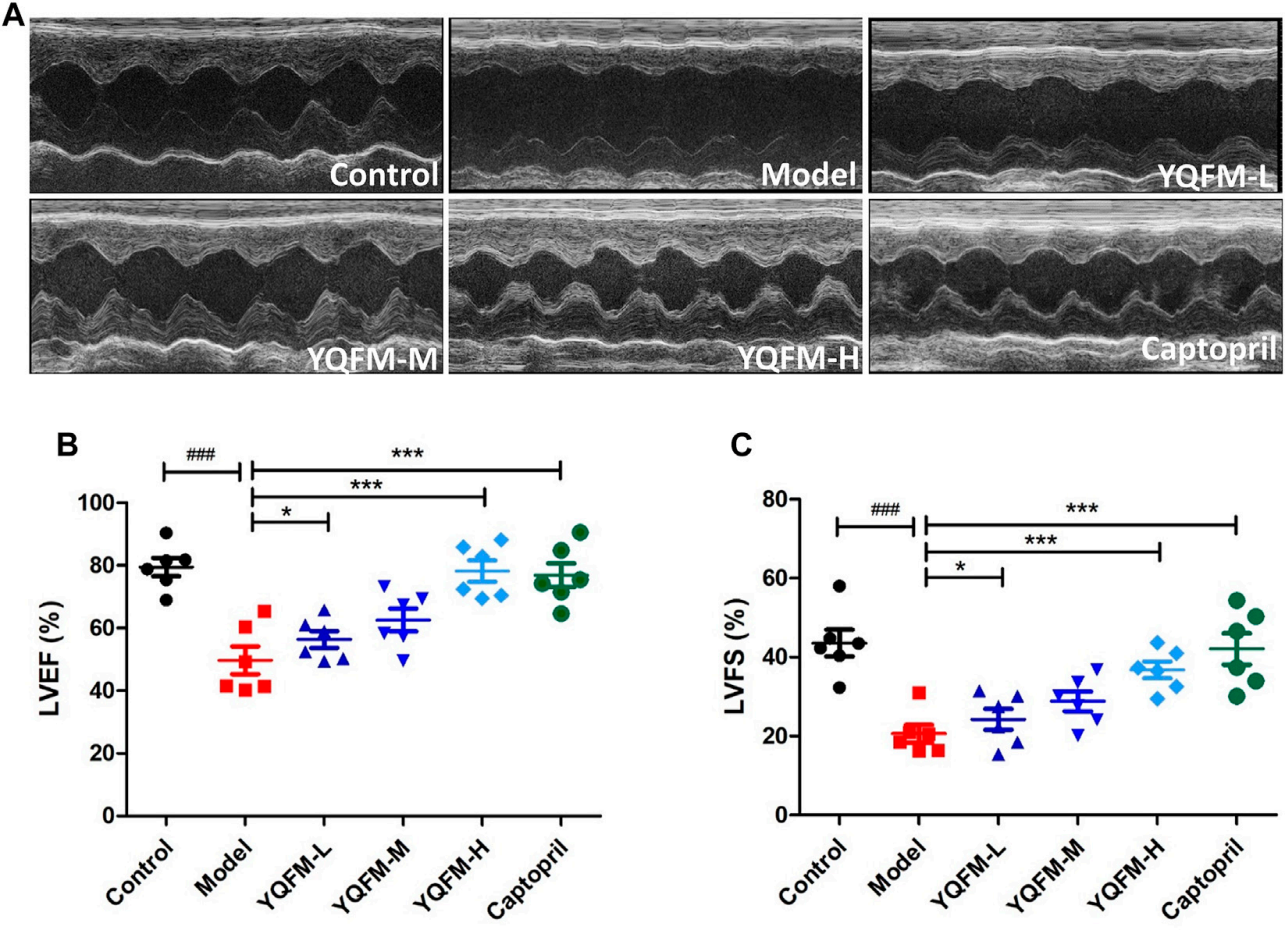
The effect of YQFM on cardiac function in CHF rat after being treated for 28 days. **(A)** Representative echocardiograph from the various groups. **(B)** Left ventricle ejection fraction (LVEF) (*n* = 6 animals/group). **(C)** Left ventricle fractional shortening (LVFS) (*n* = 6 animals/group). Data are means ± SD. ^#^ *p* < 0.05, ^##^ *p* < 0.01, ^###^ *p* < 0.001 vs the Control group, *p < 0.05, **p < 0.01, ***p < 0.001 vs the Model group.

As indicated on the Figure 2A, echocardiography was used as a method to assess cardiac function. Compared with the control group, the titers of left ventricular rejection fraction (LVEF) and left ventricular shortening fraction (LVFS) in the model group were significantly decreased by 30 and 23%, respectively, indicating that the AAC model was successfully constructed. Notably, in CHF rat administered YQFM LVEF% and LVFS% were effectively restored to normal levels, respectively (Figures 2B,C).

### Effects of Yiqi Fumai Lyophilized Injection on Cardiac Morphology in Chronic Heart Failure Rat

**FIGURE 3:**
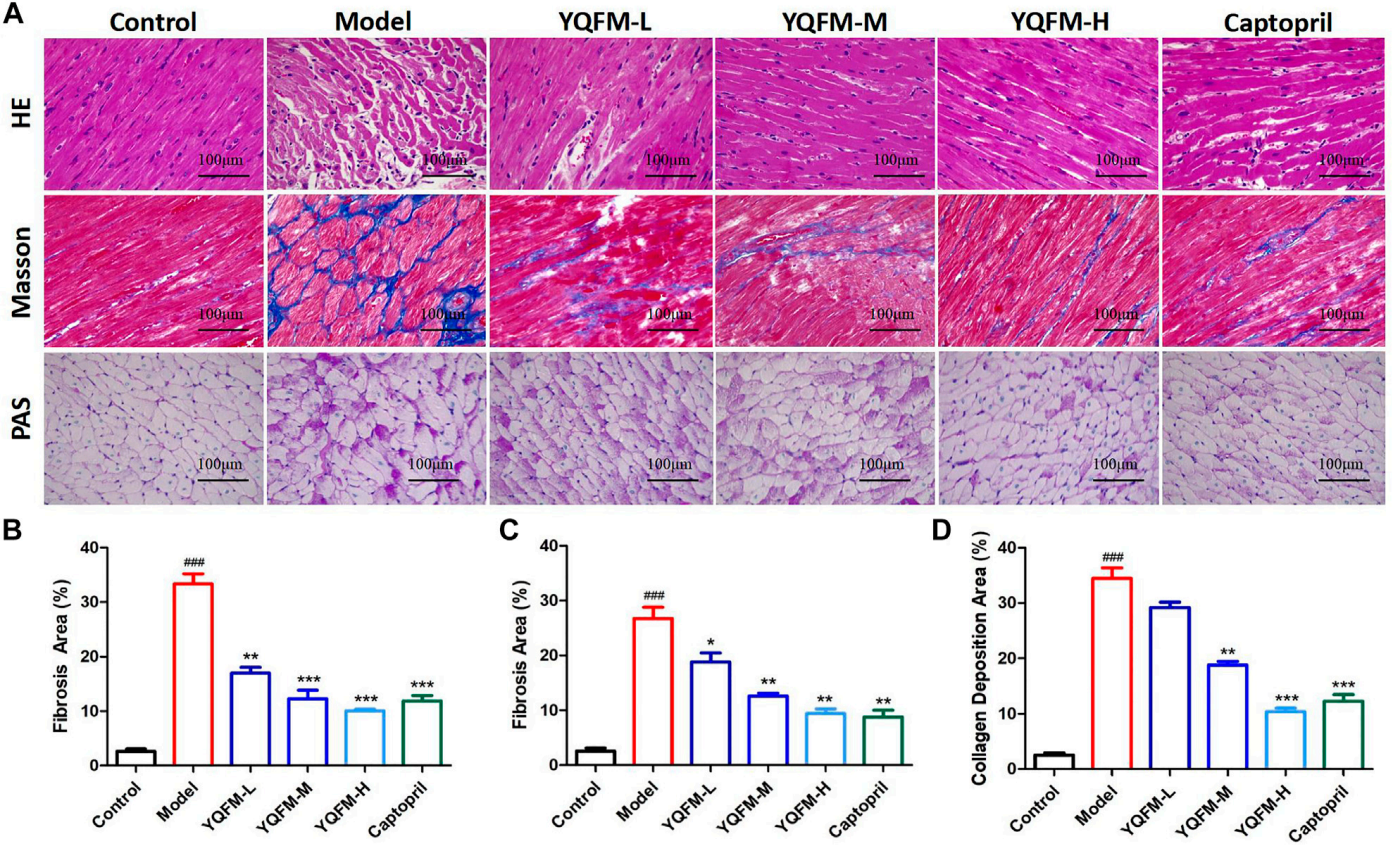
The effect of YQFM on Cardiac Morphology in CHF rat after being treated for 28 d. **(A)** HE, Masson and PAS pathology section **(B–D)** staining of the myocardial. (*n* = 6 animals/group). Data are means ± SD. ^#^ *p* < 0.05, ^##^ *p* < 0.01, ^###^ *p* < 0.001 vs the Control group, *p < 0.05, **p < 0.01, ***p < 0.001 vs the Model group.

In order to examine the therapeutic efficiency of YQFM against CHF *in vivo*, we applied YQFM to rats with cardiac dysfunction. At 28 days after treatment, the effect of YQFM was examined by histological evaluation of cardiac tissue. After YQFM treatment, myocardial cell interstitial edema, intracellular space enlargement and vacuolization, and inflammatory cell infiltration were alleviated (Figures 3A,B). Compared with the model group and captopril group, less sedimentary fiber was observed in LV as a result of YQFM treatment. It demonstrates a significantly reduced level of scar tissue (Figures 3A,C). PAS staining was consistent with Masson’s results (Figures 3A, D). Less sedimentary collagen was observed in LV after YQFM treatment.

### Effects of Yiqi Fumai Lyophilized Injection on the Biochemical Parameters of Chronic Heart Failure Rat

**FIGURE 4:**
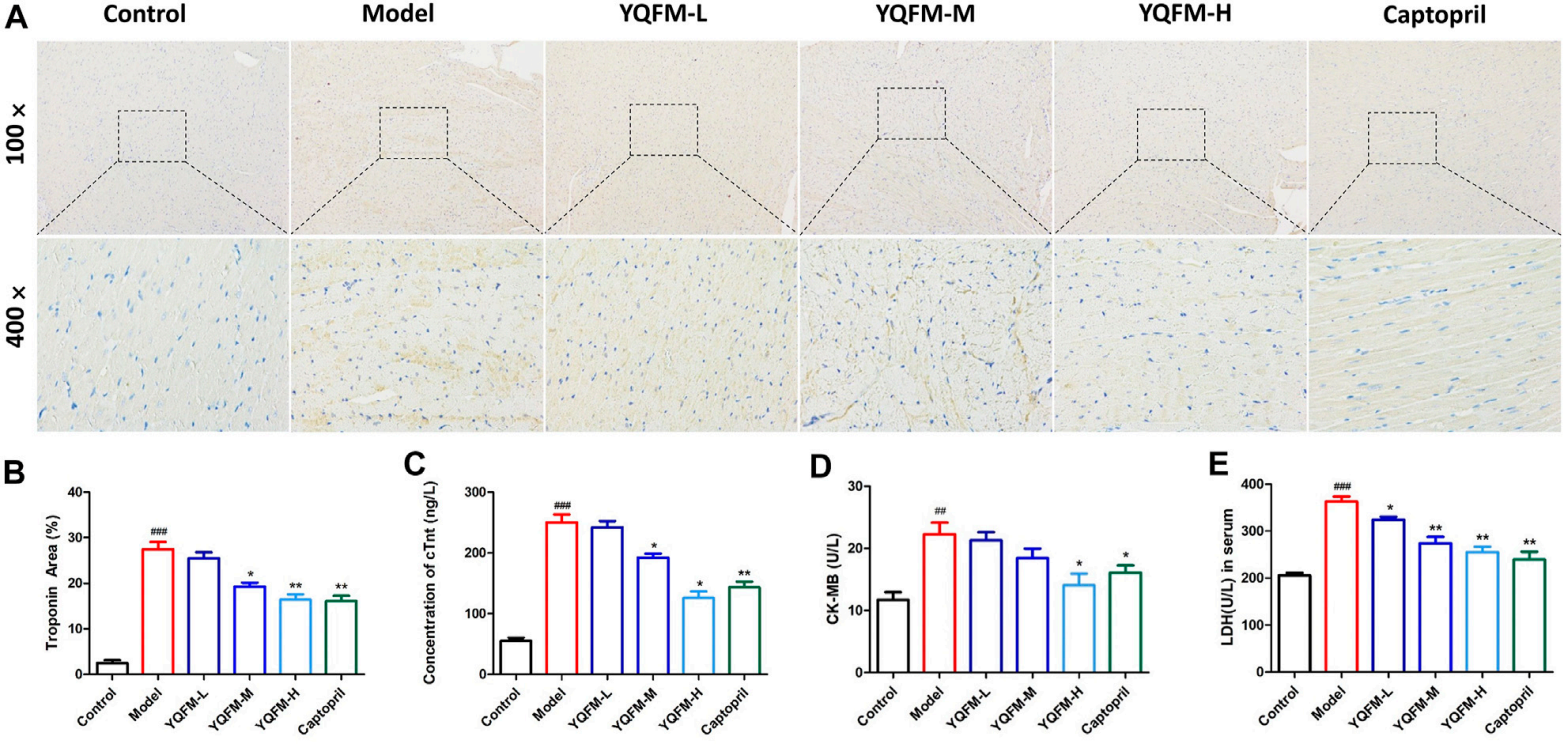
The effect of YQFM on Cardiac Morphology in CHF rat after being treated for 28 days. **(A**,**B)** Immunohistochemistry analysis of cTnt expression in heart sections of different groups. The diseased areas in the heart are magnified in the panel below (*n* = 3 per group). Concentration of cTn t **(C)**, CK-MB **(D)** and LDH **(E)** in CHF rat after being treated for 28 days (*n* = 6 animals/group). Data are means ± SD. *p < 0.05, **p < 0.01, ***p < 0.001 vs the Model group, ^#^ *p* < 0.05, ^##^ *p* < 0.01, ^###^ *p* < 0.001 vs the Control group.

To elucidate cardiac function, the cTnt titer was determined by immunohistochemical evaluation. The results are indicated in Figures 4A,B, indicating that the model groups treated by YQFM show a significant reduction. Considering the absolutely heart tissue specificity, cTnt, CK-MB, and LDH were used as the primary biometric characteristics for the diagnosis of cardiac damage. Changes in the concentrations of cTnt, CK-MB and LDH can describe the severity of myocardial injury. Our data show that cTnt, CK-MB and LDH concentrations were lower in the YQFM processing arm than in the model (AAC) group (Figures 4C–E). It implied that YQFM treatment could reduce the degree of damage and the necrosis of myocardial tissue in rats with heart failure.

### LC-MS/MS

**FIGURE 5:**
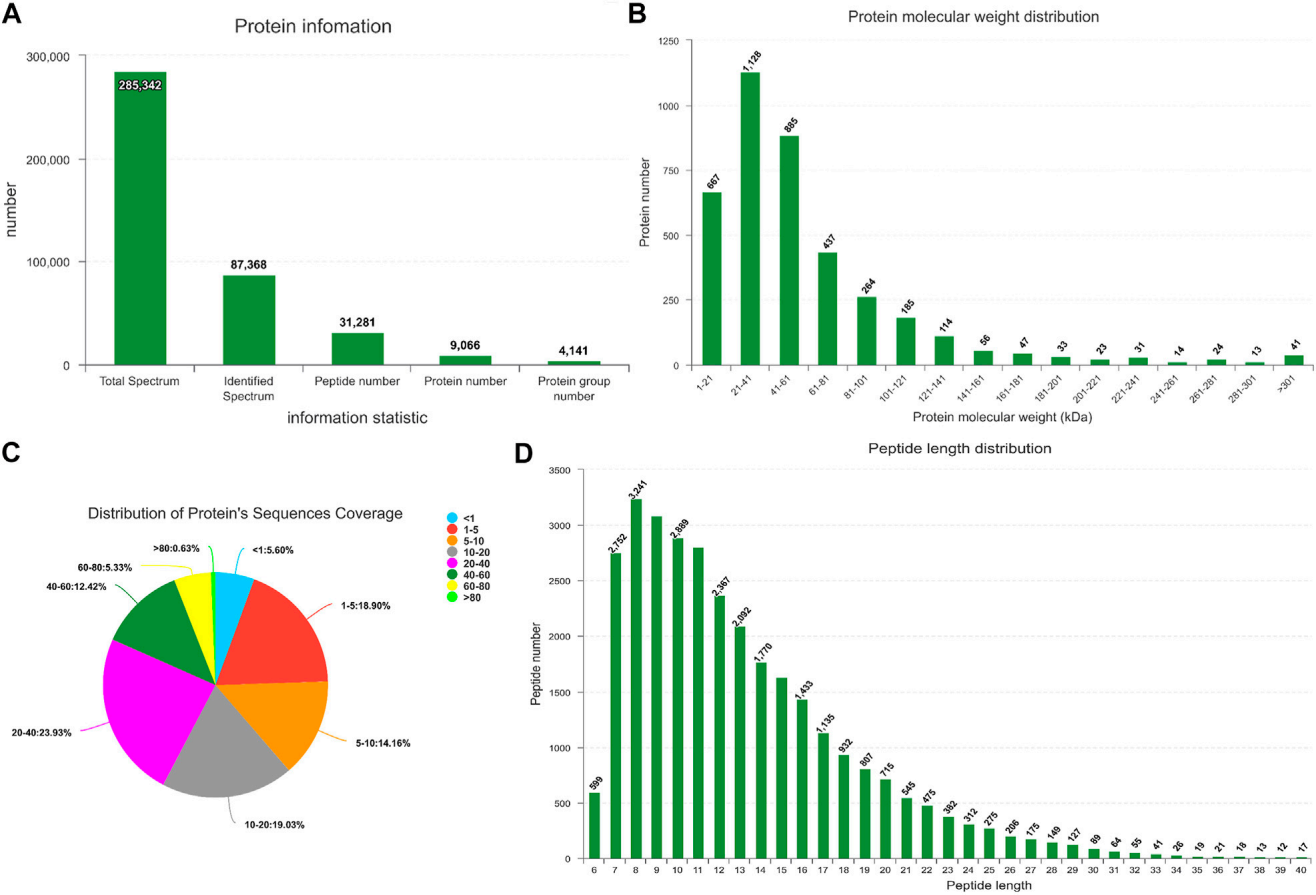
Quality control of protein data validation. **(A)** Protein information. The x-axis in the figure represents the identified protein information, from left to right: the total number of secondary spectra, the number of matched spectra, the number of identified peptides, the number of identified proteins, and the number of identified protein groups. **(B)** Protein mass distribution of all identified proteins. **(C)** Distribution of protein’s sequences coverage. **(D)** Protein length distribution of all identified peptides.

In this study, iTRAQ technology was used to analyze the proteomic characteristics of heart tissue. 9,066 proteins in total were identified (Figure 5A). The quality control of protein data revealed that the molecular weight of the protein was in the range of 1–100 kDa (Figures 5B,C), and many peptides were between 7 and 14 amino acids in length (Figure 5D).

### Identification of Differentially Expressed Proteins in Heart Tissue of Yiqi Fumai Lyophilized Injection-Treated Rat

**FIGURE 6:**
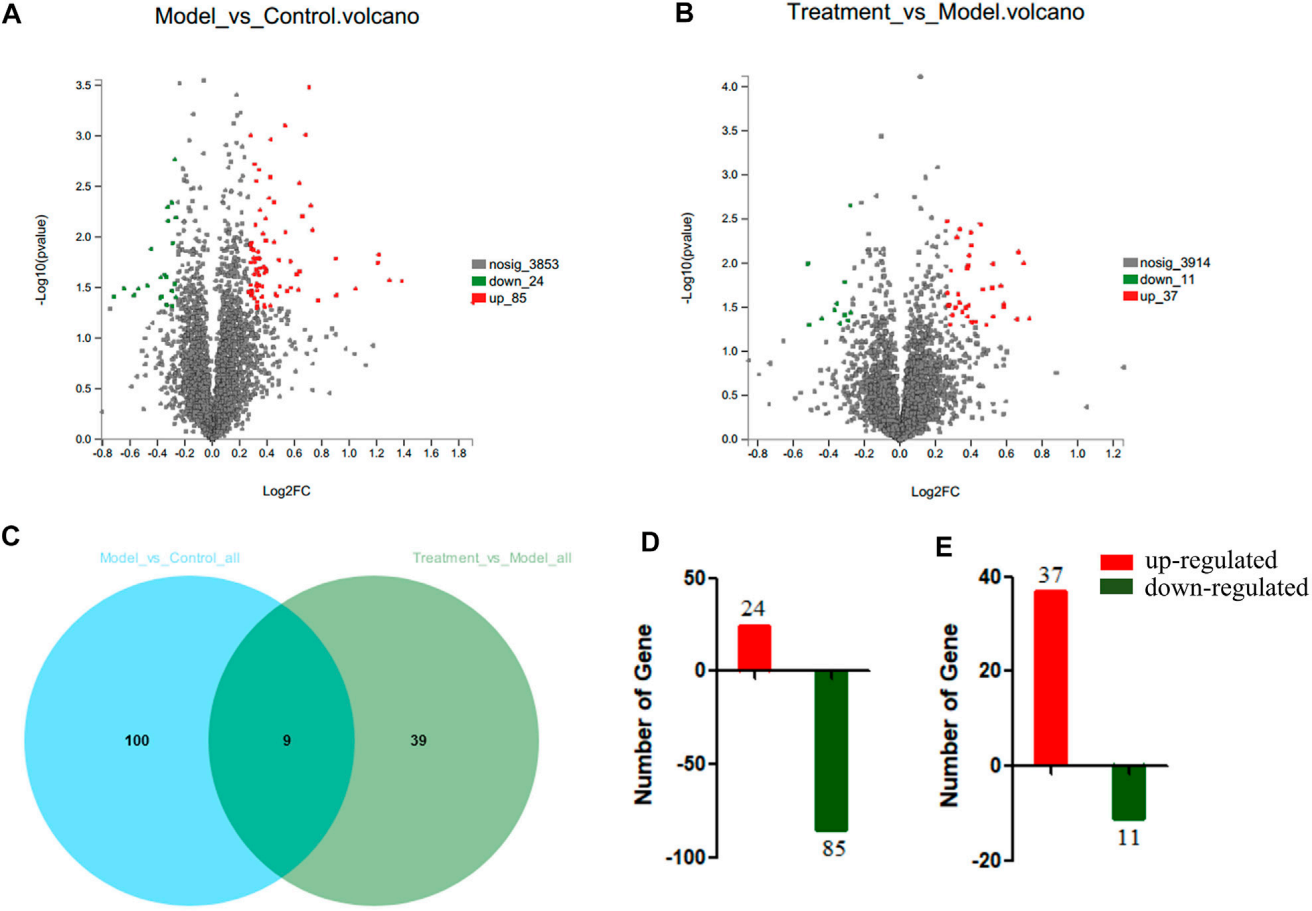
The differentially abundant proteins (DEPs) in serum between Model:Control and Treatment: Model. **(A)** Volcano Plots of DEPs of Model:Control. **(B)** Volcano Plots of DEPs of Treatment:Model. **(C)** Venn-diagram showing the overlap of all DEPs. **(D)** DEPs involved in Model:Control. **(E)** DEPs involved in Treatment:Model. Red or Green represents the numbers of upregulated and downregulated proteins, respectively.

To probe the mechanisms of YQFM, the MaxQuant was used to obtain the general summary of proteomics change in Model: Control and Treatment: Model. The ratio of Model: Control and Treatment: Model was compared to probe changes in key protein expression among the three groups (Figures 6A,B) (*p* < 0.05). 148 DEPs are displayed in a Venn diagram (Figure 6C). At the same time, 109 differentially accumulated proteins were explicated in Model: Control, 84 of which were down regulated and 24 were up regulated. 48 proteins were explicated in Treatment: Model, of which 11 were down regulated and 37 were up-regulated (Figures 6D,E).

### Clustering Analysis

**FIGURE 7:**
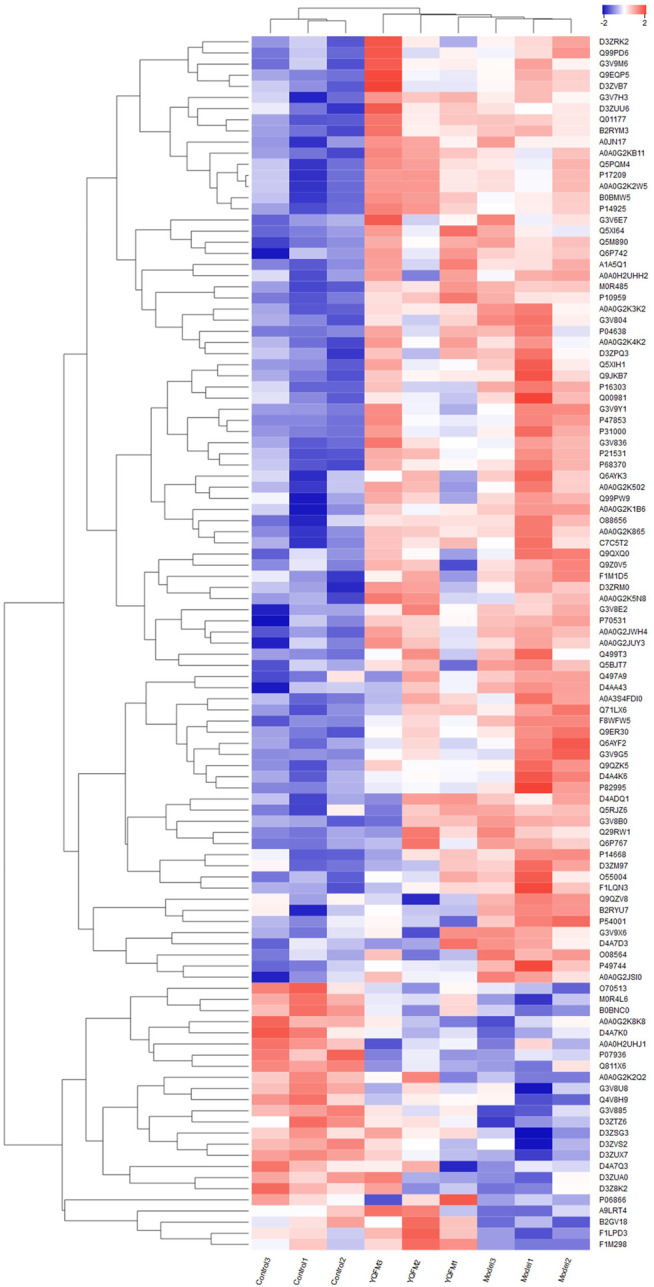
Cluster analysis of differentially expressed proteins among the three groups. Color changes with protein expression, blue indicates negative correlation, red indicates positive correlation, the stronger the color, the strongest correlation.

The results of hierarchical clustering were shown in a heat map, where blue means down regulation and red means up regulation. The difference in protein expression observed between the groups were displayed in Figure 7. It was clear that the overall protein expression pattern in the model and treatment groups was different from that in the control group. In the model group, many proteins showed a down regulated expression pattern (blue band), while in the control and treatment groups, many proteins were up regulated.

### Function Notes and Categories of Differentially Expressed Proteins

**FIGURE 8:**
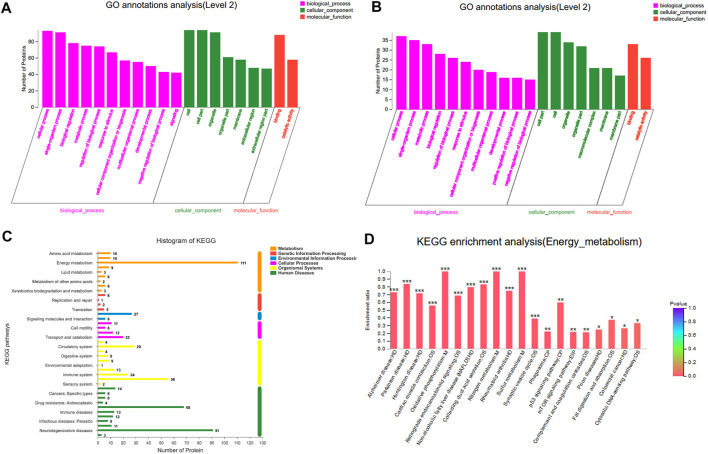
Functional annotation and categories of DEPs. **(A)** GO annotations analisis of Model:Control. **(B)** GO annotations analisis of Treatment:Model. **(C)** KEGG analyses of protein functions. **(D)** Enrichment analysis of KEGG energy metabolism pathway.

To better explore the biological correlation of protein expression changes and retrieve information about biological processes or pathways, the identified DEPs have been analyzed by bioinformatics methods. From the GO analysis results (Figures 8A,B), it can be seen that in the biological process analysis, 157 differentially expressed proteins were mainly distributed in the metabolic process, cellular process and single-organism process. In the cell location analysis among them, most proteins were mainly concentrated in the three types of cells, cell parts and organelles. As far as molecular functions were concerned, proteins related to catalytic activity and binding dominate most.

In order to further explore the biological pathway in response to YQFM protection, KEGG pathway annotation was used to explore all DEPs (Figures 8C,D). The results showed that most proteins were enriched in energy metabolism pathway. Metabolic pathway analysis was performed on the proteins in the obtained energy metabolism pathway. As shown in the figure, the oxidative phosphorylation pathway apparently plays a very important role in treatment.

### Effects of Yiqi Fumai Lyophilized Injection on H_2_O_2_ Induced Mitochondrial Dysfunction *In Vitro*

**FIGURE 9:**
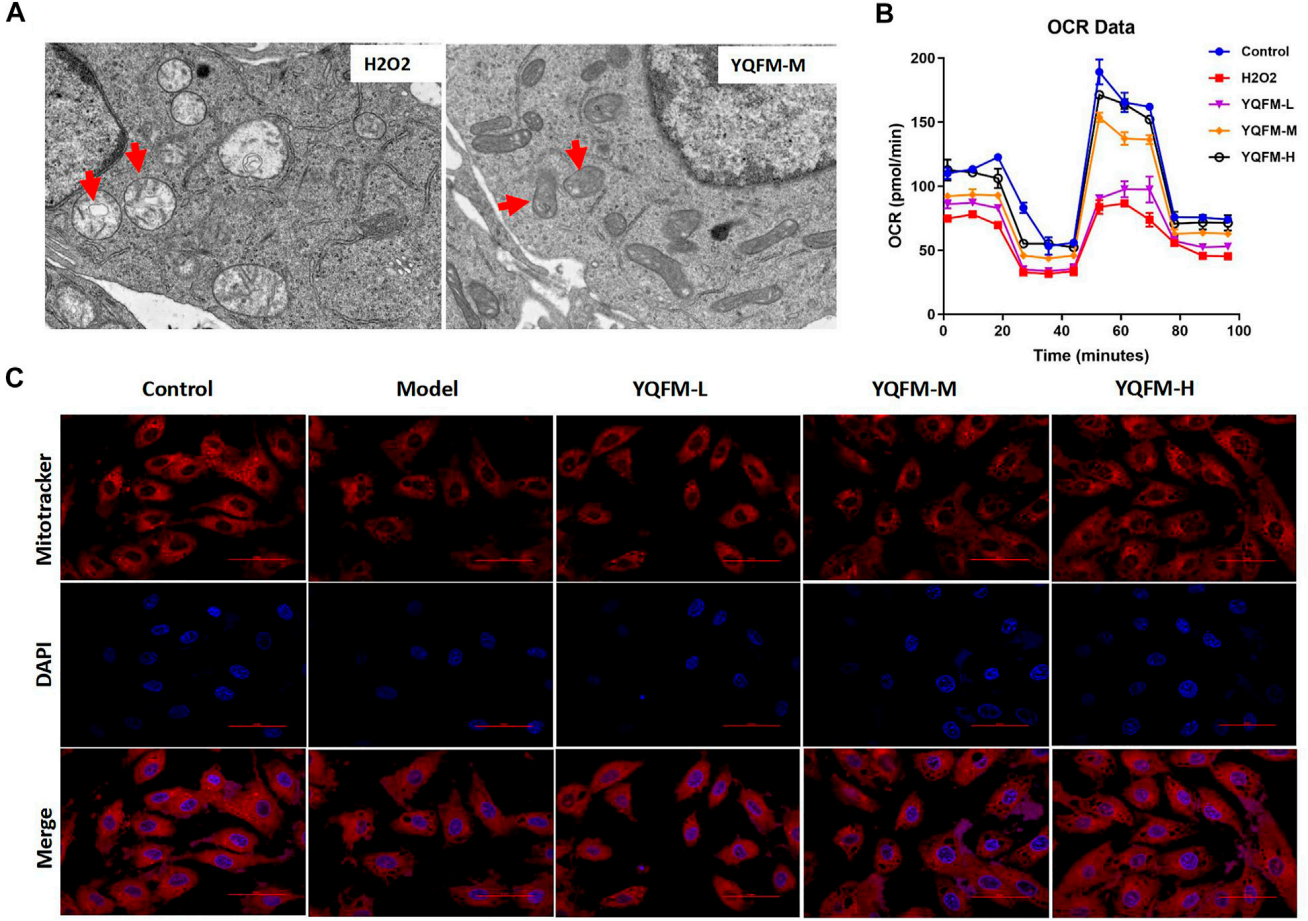
YQFM ameliorated the mitochondrial dysfunction induced by H_2_O_2_ *in vitro*. **(A)** TEM images of the ultrastructural morphology of H_9_C_2_ cells mitochondria. **(B)** Oxygen consumption rates (OCR) in H_9_C_2_ cells treated with YQFM-L, YQFM-M& YQFM-H. **(C)** H_9_C_2_ cells were subjected to Mitotracker red staining. The model group refers to H_9_C_2_ cells induced by H_2_O_2_.

Next, we investigated effects of YQFM on the mitochondrial dysfunction by using TEM, Metabolic assay, and Mitotracker staining. The ultrastructural morphology of mitochondria in H9C2 cells was observed by conventional TEM (Figures 9A,B). After hypoxia, the mitochondria of cardiomyocytes swelled, the number of mitochondria decreased, and the mitochondrial matrix appeared cristae and vacuole-like phenomena. After YQFM treatment, the damage of cardiomyocyte mitochondria was significantly reduced. We detected the effect of YQFM on OCR in H_9_C_2_ cells with H_2_O_2_ stimulation. OCR was significantly reduced in H_9_C_2_ cells stimulated by H_2_O_2_, all of which were ameliorated by YQFM. We also found that mitochondrial content of H_9_C_2_ cells was increased significantly after 24 h of YQFM treatment with 180 μg/ml, determined by the red fluorescence intensity and mitochondrial size of the mitotracker (Figure 9C). All of these results implied that YQFM ameliorated mitochondrial function in H_9_C_2_ cells stimulated by H_2_O_2_.

### Effect of Yiqi Fumai Lyophilized Injection on Apoptosis of Cardiomyocytes Induced by H_2_O_2_ *In Vitro*

**FIGURE 10:**
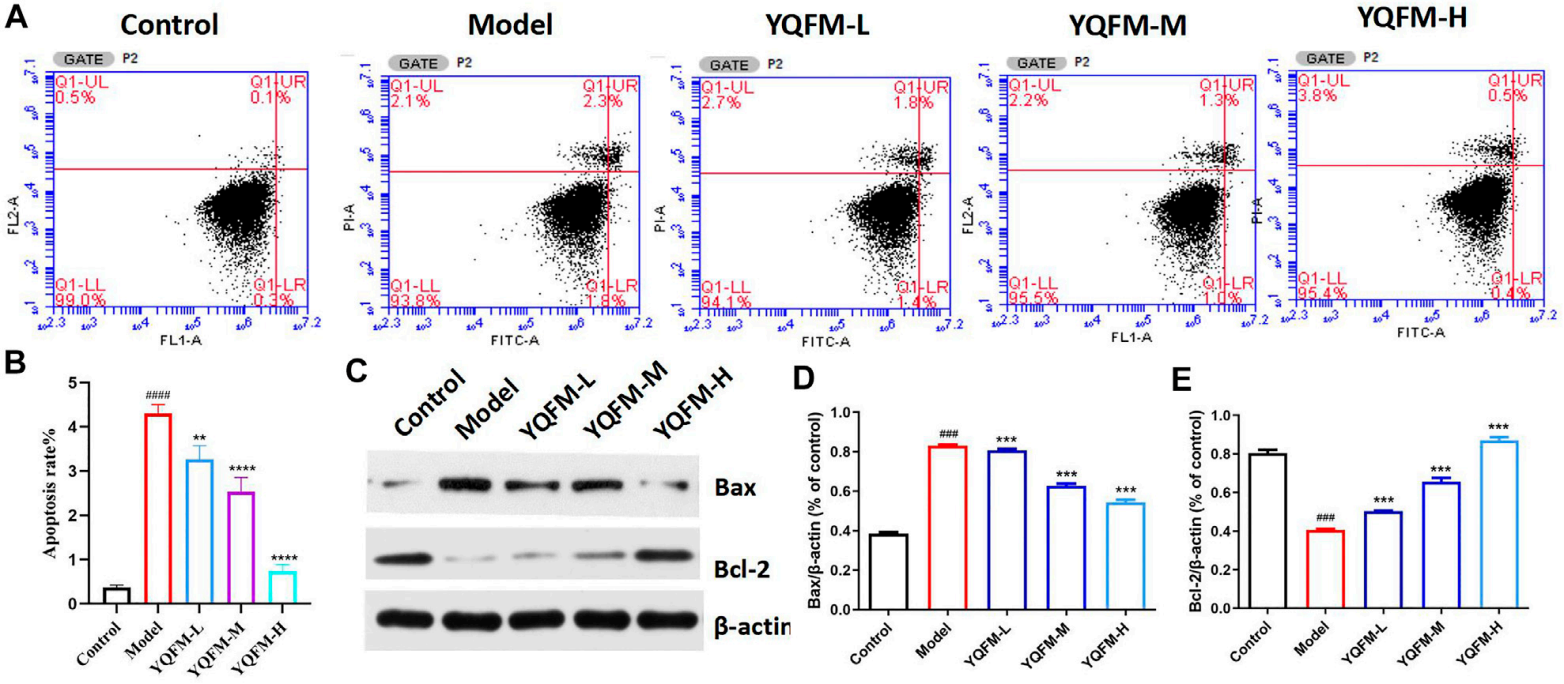
YQFM suppressed cardiomyocyte apoptosis caused by H_2_O_2_. **(A,B)** Flow cytometric measurement of H9C2 cells apoptosis caused by H2O2. **(C)** The amount of Bax and Bcl-2 from H9C2 cells lysate was examined by Western blot analysis. **(D,E)** Relative protein levels were measured by densitometry for Bax and Bcl-2 (*n* = 3 per group). Data are means ± SD. *p < 0.05, **p < 0.01, ***p < 0.001 vs the Model group, ^#^ *p* < 0.05, ^##^ *p* < 0.01, ^###^ *p* < 0.001 vs the Control group.

In the process of oxidative phosphorylation, the imbalance of intracellular ROS levels will lead to oxidative stress and ultimately lead to cell apoptosis. Therefore, we hypothesized that YQFM may improve mitochondrial function by inhibiting apoptosis. In this study, the flow cytometry and Western blot were used to evaluate the effect of YQFM on cell apoptosis. Research results indicated that YQFM significantly inhibited H2O2 induced apoptosis (Figure 10A), accompanied by decreased Bax cleavage and increased bcl-2 expression (Figures 10B–D).

### Effects of Yiqi Fumai Lyophilized Injection on Mitochondrial Biogenesis-Related Proteins *In Vitro*

**FIGURE 11:**
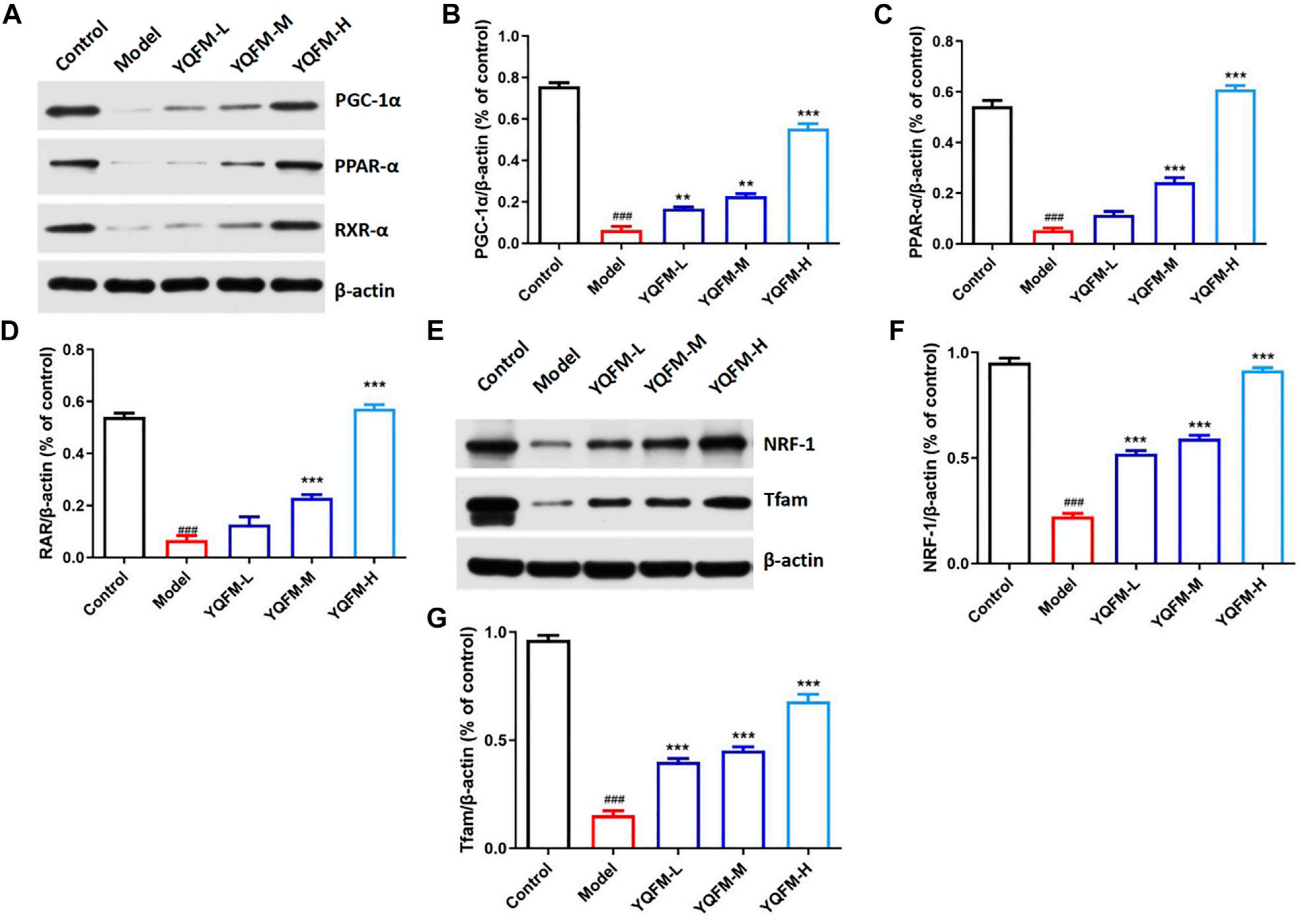
YQFM upregulated mitochondrial biogenesis-related genes with PGC-1α activation. **(A)** The amount of PGC-1α,PPAR-α and RXR-α from H9C2 cells lysate was examined by Western blot analysis. **(B–D)** Relative protein levels were measured by densitometry for PGC-1α,PPAR-α and RXR-α. **(E)** The amount of NRF-1 and Tfam from H9C2 cells lysate was examined by Western blot analysis. **(F,G)** Relative protein levels were measured by densitometry for NRF-1 and Tfam (*n* = 3 per group). Data are means ± SD. *p < 0.05, **p < 0.01, ***p < 0.001 vs the Model group, ^#^ *p* < 0.05, ^##^ *p* < 0.01, ^###^ *p* < 0.001 vs the Control group.

To determine the molecular mechanisms of mitochondrial dysfunction enhancement through YQFM treatment, we detected the expression of mitochondrial biogenic related proteins by Western blot (Figure 11A). Since previous studies have described the key role of PGC-1 α in regulating mitochondrial biogenesis, the 24-hour treatment with YQFM of 180 μg/ml can lead to a significant increase the expression of PGC-1 α. Western blot analysis further corroborated the regulatory effect of YQFM on PGC-1 α (Figure 11B). PGC-1α and PPAR-α/RXR-α jointly regulate the increase of mitochondrial activity to coordinate mitochondrial biogenesis, so YQFM-treated cardiomyocytes displayed significant increase in all of these proteins (Figures 11C,D).

In H_9_C_2_ cells treated with 180 μg/ml YQFM, NRF-1, the downstream target of PGC-1 α, was also increased at 24 h, while Tfam, the downstream target of NRF-1 and mitochondrial DNA expression regulator, had similar changes with NRF-1 (Figure 11H). These results revealed that YQFM treatment increased mitochondrial content by activating PGC-1 α and activating mitochondrial biogenesis related proteins.

## Discussion

The incidence of CHF greatly increased with the aging of the world population. (Yancy et al., 2013) Hence, seeking safe and effective therapy has become a common concern in the global medical community and has been widely concerned. Current clinical studies have indicated that YQFM could availably alleviate the clinical symptoms of CHF patients (Nie et al., 2020). At the same time, studies have shown that YQFM can improve heart failure through a variety of ways (Li et al., 2019; Zhang et al., 2019). Notwithstanding, the exact mechanism of action remains to be explored in depth. In this research, we used a proteomic based method to investigate the mechanism of YQFM in ACC model rats and on H_9_C_2_ cells, and developed a better understanding of YQFM.

CHF is a syndrome caused by the inability of the heart pump to meet the body’s energy needs (Bertero and Maack, 2018). Despite a lot of work, the pathogenesis of cardiomyocyte abnormalities caused by CHF is still not fully understood. Factors that cause abnormal contraction and relaxation of heart failure include abnormal metabolic pathways that lead to energy production, energy transfer, and reduced energy utilization (Zhou and Tian, 2018). CHF also affects the surroundings. Early muscle fatigue and motor intolerance are always present in CHF patients, in which the reduction of mitochondrial ATP production and energy transfer through phosphotransferase kinase play an important role.

Yiqi Fumai for injection is mainly extracted from the traditional Chinese medicine red ginseng, Ophiopogon japonicus and Schisandra. Its active ingredients are diverse, and it has the advantages of multiple pathways, multiple targets, and safety and effectiveness. Therefore, YQFM may also have a certain regulatory effect on myocardial energy. Studies have shown that YQFM ginsenoside Rb1, ginsenoside Rb3, ginsenoside Rg1, ginsenoside Rg3, Schizandrin B, Ophiopogonin D and other natural products can regulate energy metabolism by increasing the activity of mitochondria. In 3T3-L1 mature adipocytes, ginsenoside Rb1 can activates the mRNA expression of PGC-1α, UPC-1 and PRDM16, which in turn increased basal glucose uptake and promoted browning (Mu et al., 2015; Park et al., 2019). Ginsenoside Rb3 can regulates energy metabolism and apoptosis of cardiac myocytes by activating PPAR α pathway (Chen et al., 2019).

Ginsenoside Rg1 can protect cardiomyocytes from Hypoxia/reperfusion (H/R) by regulating GDH and MFN2 to maintain mitochondrial dynamics (Dong et al., 2016). Ginsenoside Rg3 activates PGC-1α and Nrf2 in rat myocardium, and the mRNA levels of Tfam and NRF-1 downstream of PGC-1α are enhanced (Sun et al., 2013). Schizandrin B improves mitochondrial function in damaged myocardium (Chiu et al., 2007; Chen and Ko, 2010). Ophiopogonin D intervention can reduce lipid accumulation and mitochondrial damage of heart and cardiomyocytes stimulated by palmitic acid in diabetic mice (Li et al., 2021). The enhanced mitochondrial function of these natural products is helpful to support the protective effect of YQFM on CHF.

It is well known that cTnT is the main feature of myocardial injury in CHF (Barberi and van den Hondel, 2018). The results of heart histopathology and cTn t showed that YQFM could reverse myocardial injury and alleviate CHF. In proteomics analysis, 157 DEPs was identified. GO clearly confirmed that the DEPs of Model: Control and Treatment: Model participated in various biological processes. KEGG pathway results showed that most proteins were significantly enriched in the energy metabolism pathway, especially in oxidative phosphorylation pathway.

In the process of oxidative phosphorylation, the electrons escaping from the mitochondrial electron transport chain react with oxygen to form O_2_ (Saybaşili et al., 2001).Convert it into hydrogen peroxide and other reactive oxygen species (ROS).The imbalance of intracellular ROS levels can cause oxidative stress, leading to DNA damage and eventually apoptosis. And our results demonstrate that YQFM inhibited cardiomyocyte apoptosis, improved the vacuolation state of mitochondria in cardiomyocytes, examined oxidative metabolism, and H_9_C_2_ cells mitochondrial content was significantly increased. This result further shows that YQFM can improve mitochondrial function through oxidative phosphorylation.

Mitochondria are cardinal for the survival of cardiomyocytes and the maintenance of normal heart function (Hammerling and Gustafsson, 2014). More and more evidences show that mitochondrial dysfunction can cause CHF, which indicates that there is a close relationship between mitochondrial biology and heart function (Aubert et al., 2013; Wang et al., 2015b; Tao et al., 2015). Studies have shown that PGC-1α is a key molecule of mitochondria, and PGC-1α is involved in mitochondrial energy metabolism and plays a key role in oxidative stress and inflammation (Jeganathan et al., 2017). PGC-1α is an important synergistic factor of PPAR/RXR (Warren et al., 2018), which regulates the metabolism of lipids and sugars by acting with PPAR/RXR. In addition to its action with PPARs, PGC-1α mainly binds to two other transcription factors to regulate the function of cardiac mitochondria. That is, activation of PGC-1α protein can activate downstream transcription factors like NRFs and Tfam, thereby promoting mitochondrial biosynthesis. At the same time, it promotes physiological processes such as glucose utilization and fatty acid oxidation (Huang et al., 2017). In our study, the expressions of PGC-1α, PPAR-α and RXR-α were up regulated in cells treated with YQFM for 180 μg/ml for 24 h, and PGC-1 α and its downstream effectors, including NRF-1 and TFAM, were also found to be up regulated in cardiac myocytes.

These results provide evidence that YQFM could enhance mitochondrial function and improve mitochondrial energy metabolism of cardiomyocytes by regulating PGC-1α and its related proteins.

## Conclusion

In summary, we performed proteomic characteristics of heart tissue analysis of protein expression in heart tissue of AAC rats treated with YQFM using an iTRAQ technology. A total of 157 important DEPs were identified, including 109 in M: C and 48 in T: M. Intensive bioinformatics analysis identified metabolic process, cellular process and single-organism process as significant biological processes. The oxidative phosphorylation process were mainly involved, especially the mitochondrial PGC-1α signaling pathway. In general, the results of this study were helpful to understand of the mechanisms of YQFM. In addition, this work demonstrated the potential application of the iTRAQ technique in Anti-CHF studies.

## Data Availability

The datasets presented in this study can be found in online repositories. The names of the repository/repositories and accession number(s) can be found below: ProteomeXchange (https://www.ebi.ac.uk/pride/archive/login) via the PRIDE repository with the dataset identifier PXD027442.

## Abbreviations

AAC, Abdominal aortic coarctation; Bax, Bcl-2-associated X protein; Bcl-2, B-cell lymphoma-2; CHF, Chronic heart failure; DEPs, Differentially expressed proteins; GO, Gene Ontology; HE, hematoxylin and eosin; H_2_O_2_, hydrogen peroxide; KEGG, Kyoto Encyclopedia of Genes and Genome; LVEF, left ventricular rejection fraction; LVFS, left ventricular fractional Shortening; NRF-1, nuclear respiratory factor 1; PAS, Periodic Acid Schiff; PGC-1α, Peroxisome proliferator-activated receptor-γ coactivator-1α; PPAR-α, perixisome proliferation-activated receptor alpha; RXR-α, retinoid X receptor alpha; SMS, Sheng mai san; TCM, Traditional Chinese medicine; TEM, Transmission Electron Microscope; Tfam, mitochondrial transcription factor A; TMT, tandem mass tags; YQFM, Yiqi Fumai lyophilized injection.

## References

[B1] Aubert G. Vega R. B. Kelly D. P. (2013). Perturbations in the Gene Regulatory Pathways Controlling Mitochondrial Energy Production in the Failing Heart. Biochim. Biophys. Acta 1833, 840–847. 10.1016/j.bbamcr.2012.08.015 22964268 PMC3570640

[B2] Barberi C. van den Hondel K. E. (2018). The Use of Cardiac Troponin T (cTnT) in the Postmortem Diagnosis of Acute Myocardial Infarction and Sudden Cardiac Death: A Systematic Review. Forensic Sci. Int. 292, 27–38. 10.1016/j.forsciint.2018.09.002 30269044

[B3] Bertero E. Maack C. (2018). Metabolic Remodelling in Heart Failure. Nat. Rev. Cardiol. 15, 457–470. 10.1038/s41569-018-0044-6 29915254

[B4] Bertinchant J. P. Robert E. Polge A. Marty-Double C. Fabbro-Peray P. Poirey S. (2000). Comparison of the Diagnostic Value of Cardiac Troponin I and T Determinations for Detecting Early Myocardial Damage and the Relationship with Histological Findings after Isoprenaline-Induced Cardiac Injury in Rats. Clin. Chim. Acta 298, 13–28. 10.1016/s0009-8981(00)00223-0 10876001

[B5] Boersema P. J. Raijmakers R. Lemeer S. Mohammed S. Heck A. J. (2009). Multiplex Peptide Stable Isotope Dimethyl Labeling for Quantitative Proteomics. Nat. Protoc. 4, 484–494. 10.1038/nprot.2009.21 19300442

[B6] Chen N. Ko M. (2010). Schisandrin B-Induced Glutathione Antioxidant Response and Cardioprotection Are Mediated by Reactive Oxidant Species Production in Rat Hearts. Biol. Pharm. Bull. 33, 825–829. 10.1248/bpb.33.825 20460761

[B7] Chen X. Wang Q. Shao M. Ma L. Guo D. Wu Y. (2019). Ginsenoside Rb3 Regulates Energy Metabolism and Apoptosis in Cardiomyocytes via Activating PPARα Pathway. Biomed. Pharmacother. 120, 109487. 10.1016/j.biopha.2019.109487 31577975

[B8] Chiu P. Y. Leung H. Y. Siu A. H. Poon M. K. Ko K. M. (2007). Schisandrin B Decreases the Sensitivity of Mitochondria to Calcium Ion-Induced Permeability Transition and Protects against Ischemia-Reperfusion Injury in Rat Hearts. Acta Pharmacol. Sin 28, 1559–1565. 10.1111/j.1745-7254.2007.00614.x 17883940

[B9] Cops J. Haesen S. De Moor B. Mullens W. Hansen D. (2019). Current Animal Models for the Study of Congestion in Heart Failure: an Overview. Heart Fail. Rev. 24, 387–397. 10.1007/s10741-018-9762-4 30612214 PMC6476831

[B10] Dong G. Chen T. Ren X. Zhang Z. Huang W. Liu L. (2016). Rg1 Prevents Myocardial Hypoxia/reoxygenation Injury by Regulating Mitochondrial Dynamics Imbalance via Modulation of Glutamate Dehydrogenase and Mitofusin 2. Mitochondrion 26, 7–18. 10.1016/j.mito.2015.11.003 26593335

[B11] Faris R. Flather M. D. Purcell H. Poole-Wilson P. A. Coats A. J. (2012). Diuretics for Heart Failure. Cochrane Database Syst. Rev. (2), CD003838. 10.1002/14651858.CD003838 22336795

[B12] Feng Y. Q. Ju A. C. Liu C. H. Wang T. Yu B. Y. Qi J. (2016). Protective Effect of the Extract of Yi-Qi-Fu-Mai Preparation on Hypoxia-Induced Heart Injury in Mice. Chin. J. Nat. Med. 14, 401–406. 10.1016/S1875-5364(16)30035-8 27473956

[B13] Fu S. Zhang J. Gao X. Xia Y. Ferrelli R. Fauci A. (2010). Clinical Practice of Traditional Chinese Medicines for Chronic Heart Failure. Heart Asia 2, 24–27. 10.1136/ha.2009.001123 27325938 PMC4898503

[B14] Giannitsis E. Katus H. A. (2013). Cardiac Troponin Level Elevations Not Related to Acute Coronary Syndromes. Nat. Rev. Cardiolcardiology 10, 623–634. 10.1038/nrcardio.2013.129 23979214

[B15] Hammerling B. C. Gustafsson Å. B. (2014). Mitochondrial Quality Control in the Myocardium: Cooperation between Protein Degradation and Mitophagy. J. Mol. Cel Cardiol 75, 122–130. 10.1016/j.yjmcc.2014.07.013 PMC4159946 25086292

[B16] Heo S. Doering L. V. Widener J. Moser D. K. (2008). Predictors and Effect of Physical Symptom Status on Health-Related Quality of Life in Patients with Heart Failure. Am. J. Crit. Care 17, 124–132. 10.4037/ajcc2008.17.2.124 18310649

[B17] Huang T. Y. Zheng D. Houmard J. A. Brault J. J. Hickner R. C. Cortright R. N. (2017). Overexpression of PGC-1α Increases Peroxisomal Activity and Mitochondrial Fatty Acid Oxidation in Human Primary Myotubes. Am. J. Physiol. Endocrinol. Metab. 312, E253–E263. 10.1152/ajpendo.00331.2016 28073778 PMC5406987

[B18] Jeganathan J. Saraf R. Mahmood F. Pal A. Bhasin M. K. Huang T. (2017). Mitochondrial Dysfunction in Atrial Tissue of Patients Developing Postoperative Atrial Fibrillation. Ann. Thorac. Surg. 104, 1547–1555. 10.1016/j.athoracsur.2017.04.060 28760472

[B19] Li F. Pang L. Z. Zhang L. Zhang Y. Zhang Y. Y. Yu B. Y. (2019). YiQiFuMai Powder Injection Ameliorates Chronic Heart Failure through Cross-Talk between Adipose Tissue and Cardiomyocytes via Up-Regulation of Circulating Adipokine Omentin. Biomed. Pharmacother. 119, 109418. 10.1016/j.biopha.2019.109418 31505423

[B20] Li F. Zheng X. Fan X. Zhai K. Tan Y. Kou J. (2016). YiQiFuMai Powder Injection Attenuates Ischemia/Reperfusion-Induced Myocardial Apoptosis through AMPK Activation. Rejuvenation Res. 19, 495–508. 10.1089/rej.2015.1801 27072567

[B21] Li W. Ji L. Tian J. Tang W. Shan X. Zhao P. (2021). Ophiopogonin D Alleviates Diabetic Myocardial Injuries by Regulating Mitochondrial Dynamics. J. Ethnopharmacol. 271, 113853. 10.1016/j.jep.2021.113853 33485986

[B22] Liu J. Xu B. Liu Z. Dong M. Mao J. Zhou Y. (2017). Specific Mixing Facilitates the Comparative Quantification of Phosphorylation Sites with Significant Dysregulations. Anal. Chim. Acta 950, 129–137. 10.1016/j.aca.2016.10.044 27916117

[B23] Luo T. Chen B. Wang X. (2015). 4-PBA Prevents Pressure Overload-Induced Myocardial Hypertrophy and Interstitial Fibrosis by Attenuating Endoplasmic Reticulum Stress. Chem. Biol. Interact 242, 99–106. 10.1016/j.cbi.2015.09.025 26428355 PMC4695313

[B24] Mosterd A. Hoes A. W. (2007). Clinical Epidemiology of Heart Failure. Heart 93, 1137–1146. 10.1136/hrt.2003.025270 17699180 PMC1955040

[B25] Motoyama A. Yates J. R. 3rd (2008). Multidimensional LC Separations in Shotgun Proteomics. Anal. Chem. 80, 7187–7193. 10.1021/ac8013669 18826178

[B26] Mu Q. Fang X. Li X. Zhao D. Mo F. Jiang G. (2015). Ginsenoside Rb1 Promotes browning through Regulation of PPARγ in 3T3-L1 Adipocytes. Biochem. Biophys. Res. Commun. 466, 530–535. 10.101610.1016/j.bbrc.2015.09.064 26381176

[B27] Nie H. Li S. Liu M. Zhu W. Zhou X. Yan D. (2020). Yiqi Fumai Injection as an Adjuvant Therapy in Treating Chronic Heart Failure: A Meta-Analysis of 33 Randomized Controlled Trials. Evid. Based Complement. Alternat Medecam 2020, 1876080. 10.1155/2020/1876080 PMC7453275 32922505

[B28] Pang L. Z. Ju A. C. Zheng X. J. Li F. Song Y. F. Zhao Y. (2017). YiQiFuMai Powder Injection Attenuates Coronary Artery Ligation-Induced Myocardial Remodeling and Heart Failure through Modulating MAPKs Signaling Pathway. J. Ethnopharmacol. 202, 67–77. 10.1016/j.jep.2017.02.032 28237302

[B29] Park S. J. Park M. Sharma A. Kim K. Lee H. J. (2019). Black Ginseng and Ginsenoside Rb1 Promote Browning by Inducing UCP1 Expression in 3T3-L1 and Primary White Adipocytes. Nutrients 11, 2747. 10.3390/nu11112747 PMC6893667 31726767

[B30] Saybaşili H. Yüksel M. Haklar G. Yalçin A. S. (2001). Effect of Mitochondrial Electron Transport Chain Inhibitors on Superoxide Radical Generation in Rat Hippocampal and Striatal Slices. Antioxid. Redox Signaling 3, 1099–1104. 10.1089/152308601317203602 11813983

[B31] Song C. Wang F. Ye M. Cheng K. Chen R. Zhu J. (2011). Improvement of the Quantification Accuracy and Throughput for Phosphoproteome Analysis by a Pseudo Triplex Stable Isotope Dimethyl Labeling Approach. Anal. Chem. 83, 7755–7762. 10.1021/ac201299j 21902226

[B32] Sun M. Huang C. Wang C. Zheng J. Zhang P. Xu Y. (2013). Ginsenoside Rg3 Improves Cardiac Mitochondrial Population Quality: Mimetic Exercise Training. Biochem. Biophys. Res. Commun. 441, 169–174. 10.1016/j.bbrc.2013.10.039 24140059

[B33] Suo T. Wang H. Li Z. (2016). Application of Proteomics in Research on Traditional Chinese Medicine. Expert Rev. Proteom. 13, 873–881. 10.1080/14789450.2016.1220837 27488052

[B34] Tao L. Bei Y. Lin S. Zhang H. Zhou Y. Jiang J. (2015). Exercise Training Protects against Acute Myocardial Infarction via Improving Myocardial Energy Metabolism and Mitochondrial Biogenesis. Cell Physiol. Biochem. 37, 162–175. 10.1159/000430342 26303678

[B35] Wang F. Chen R. Zhu J. Sun D. Song C. Wu Y. (2010). A Fully Automated System with Online Sample Loading, Isotope Dimethyl Labeling and Multidimensional Separation for High-Throughput Quantitative Proteome Analysis. Anal. Chem. 82, 3007–3015. 10.1021/ac100075y 20230046

[B36] Wang H. Bei Y. Lu Y. Sun W. Liu Q. Wang Y. (2015). Exercise Prevents Cardiac Injury and Improves Mitochondrial Biogenesis in Advanced Diabetic Cardiomyopathy with PGC-1α and Akt Activation. Cel Physiol Biochem 35, 2159–2168. 10.1159/000374021 25896313

[B37] Wang H. Z. Tian J. B. Yang K. H. (2015). Efficacy and Safety of LCI699 for Hypertension: a Meta-Analysis of Randomized Controlled Trials and Systematic Review. Eur. Rev. Med. Pharmacol. Sci. 19, 296–304. 25683946

[B38] Wang Q. Dong L. Jian Z. Tang X. (2017). Effectiveness of a PRECEDE-Based Education Intervention on Quality of Life in Elderly Patients with Chronic Heart Failure. BMC Cardiovasc. Disord. 17, 262. 10.1186/s12872-017-0698-8 29037148 PMC5644077

[B39] Warren J. S. Tracy C. M. Miller M. R. Makaju A. Szulik M. W. Oka S. I. (2018). Histone Methyltransferase Smyd1 Regulates Mitochondrial Energetics in the Heart. Proc. Natl. Acad. Sci. U S A. 115, E7871–E7880. 10.1073/pnas.1800680115 30061404 PMC6099878

[B40] Wei J. Guo F. Zhang M. Xian M. Wang T. Gao J. (2019). Signature-oriented Investigation of the Efficacy of Multicomponent Drugs against Heart Failure. FASEB J. 33, 2187–2198. 10.1096/fj.201800673RR 30230922

[B41] Wilhelm M. Schlegl J. Hahne H. Gholami A. M. Lieberenz M. Savitski M. M. (2014). Mass-spectrometry-based Draft of the Human Proteome. Nature 509, 582–587. 10.1038/nature13319 24870543

[B42] Wiśniewski J. R. Zougman A. Nagaraj N. Mann M. (2009). Universal Sample Preparation Method for Proteome Analysis. Nat. Methods 6, 359–362. 10.1038/nmeth.1322 19377485

[B43] Xing L. Jiang M. Dong L. Gao J. Hou Y. Bai G. (2013). Cardioprotective Effects of the YiQiFuMai Injection and Isolated Compounds on Attenuating Chronic Heart Failure via NF-Κb Inactivation and Cytokine Suppression. J. Ethnopharmacol 148, 239–245. 10.1016/j.jep.2013.04.019 23619019

[B44] Xu B. Wang F. Song C. Sun Z. Cheng K. Tan Y. (2014). Large-scale Proteome Quantification of Hepatocellular Carcinoma Tissues by a Three-Dimensional Liquid Chromatography Strategy Integrated with Sample Preparation. J. Proteome Res. 13, 3645–3654. 10.1021/pr500200s 24972180

[B45] Yancy C. W. Jessup M. Bozkurt B. Butler J. Casey D. E. Drazner M. H. (2013). 2013 ACCF/AHA Guideline for the Management of Heart Failure: Executive Summary: a Report of the American College of Cardiology Foundation/American Heart Association Task Force on Practice Guidelines. Circulation 128, 1810–1852. 10.1016/j.jacc.2013.05.019 23741057

[B46] Zhang Y. Zhang L. Zhang Y. Fan X. Yang W. Yu B. (2019). YiQiFuMai Powder Injection Attenuates Coronary Artery Ligation-Induced Heart Failure through Improving Mitochondrial Function via Regulating ROS Generation and CaMKII Signaling Pathways. Front. Pharmacol. 10, 381. 10.3389/fphar.2019.00381 31031629 PMC6470332

[B47] Zheng H. R. Chu Y. Zhou D. Z. Ju A. C. Li W. Li X. (2018). Integrated Pharmacokinetics of Ginsenosides after Intravenous Administration of YiQiFuMai Powder Injection in Rats with Chronic Heart Failure by UFLC-MS/MS. J. Chromatogr. B Analyt Technol. Biomed. Life Sci. 1072, 282–289. 10.1016/j.jchromb.2017.10.056 29202359

[B48] Zhou B. Tian R. (2018). Mitochondrial Dysfunction in Pathophysiology of Heart Failure. J. Clin. Invest. 128, 3716–3726. 10.1172/JCI120849 30124471 PMC6118589

