# Supplementary material for: Proteomic Analysis Reveals the Protective Effects of Yiqi Fumai Lyophilized Injection on Chronic Heart Failure by Improving Myocardial Energy Metabolism
Source: Front Pharmacol. 2021 Sep 21;12:719532. doi: 10.3389/fphar.2021.719532 (PMC8494180; doi:10.3389/fphar.2021.719532)
Supplement: Supplementary file 1 [file DataSheet1.PDF]

panel A

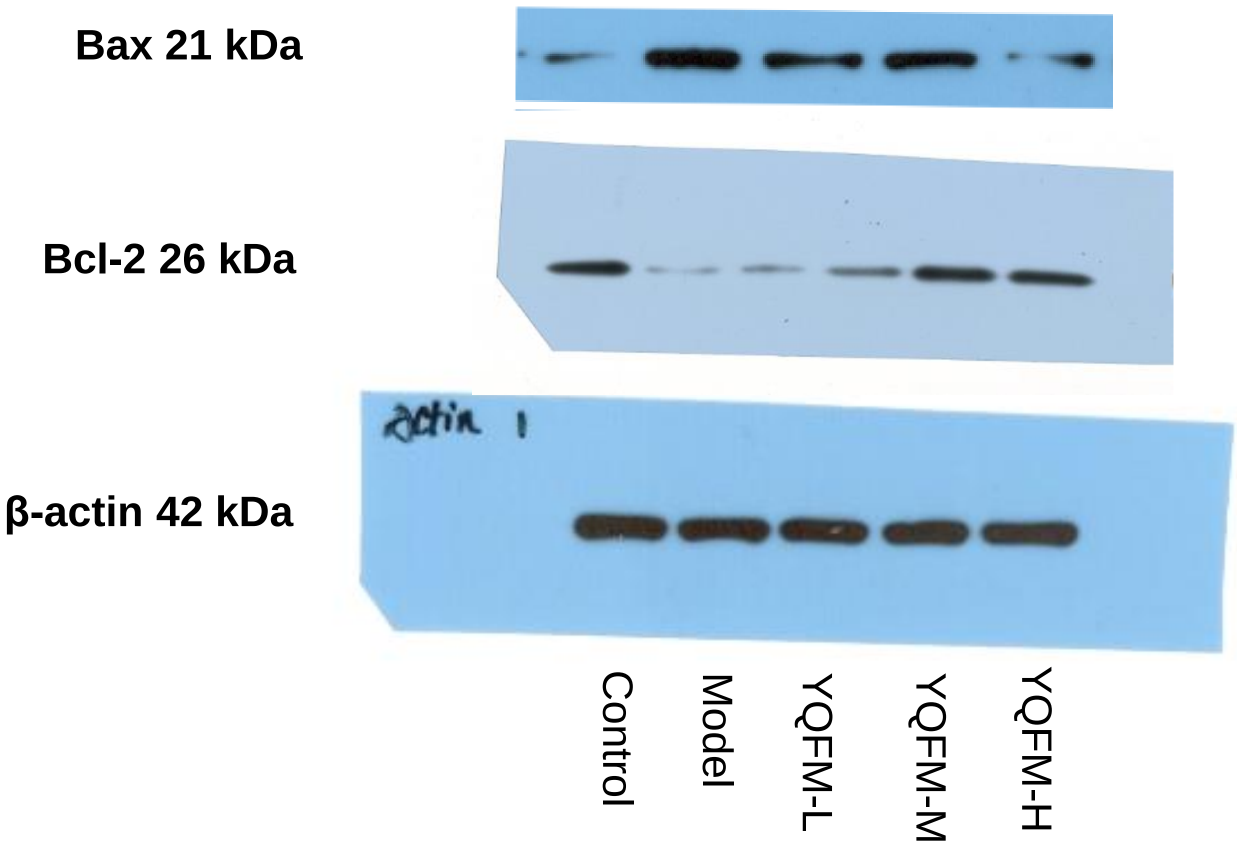

panel A represents western blot analysis shown in Fig 10.

Figure 10 shows the whole blot for Bax (21 kDa) and Bcl-2(26 kDa).

panel B

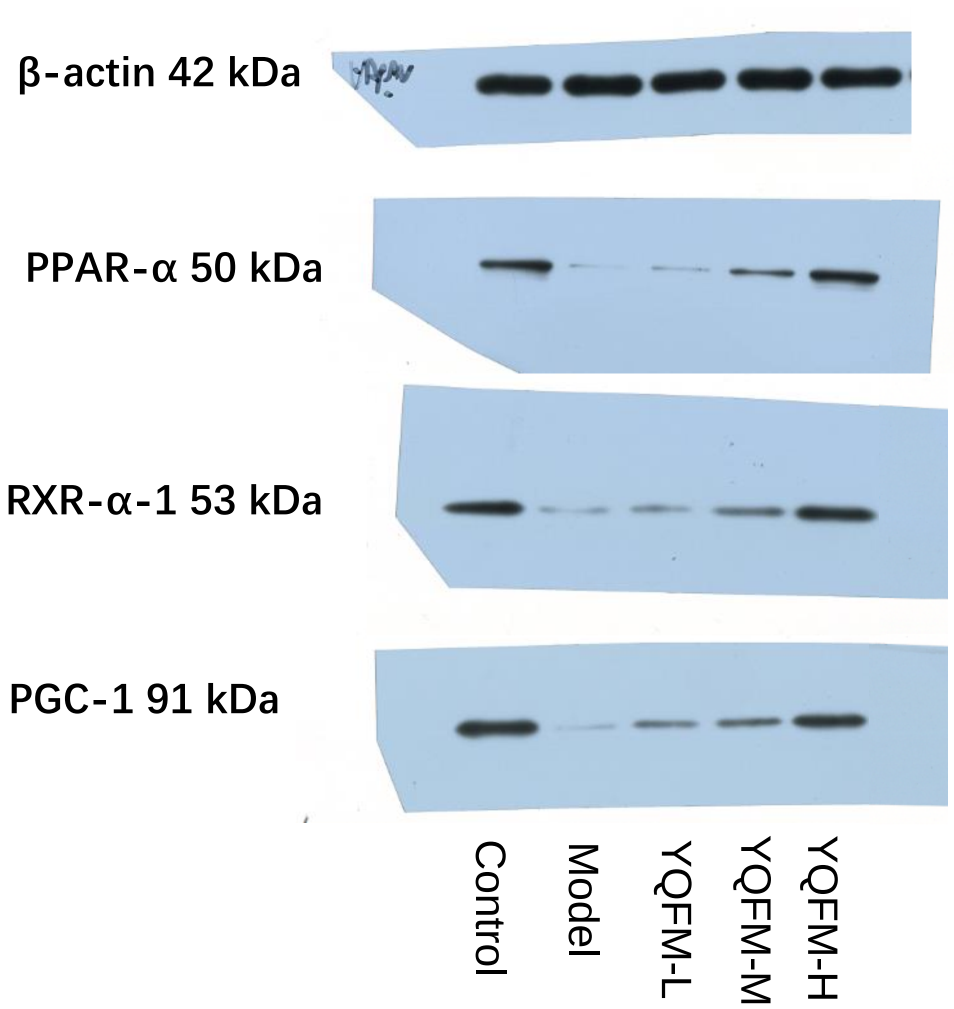

panel B represents western blot analysis shown in Fig 11.

Figure 11 shows the whole blot for PPAR-α (50 kDa), RXR-α-1 (53 kDa) and PGC-1 (91 kDa).

panel C

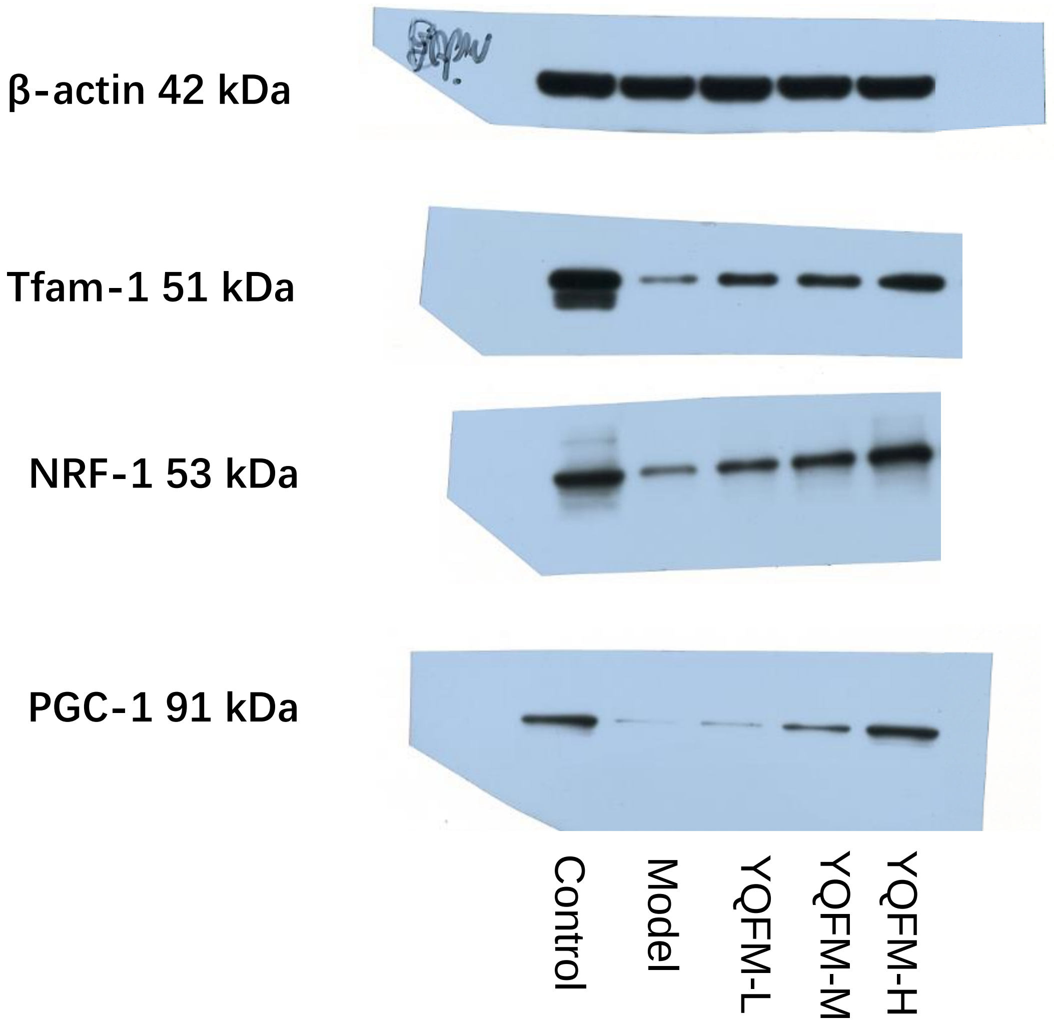

panel C represents western blot analysis shown in Fig 11.

Figure 11 shows the whole blot for Tfam-1 (51 kDa) , NRF-1 (53 kDa)and PGC-1 (91 kDa).
